# Features and structure of a cold active *N*-acetylneuraminate lyase

**DOI:** 10.1371/journal.pone.0217713

**Published:** 2019-06-11

**Authors:** Man Kumari Gurung, Bjørn Altermark, Ronny Helland, Arne O. Smalås, Inger Lin U. Ræder

**Affiliations:** The Norwegian Structural Biology Center (NorStruct), Department of Chemistry, UiT- The Arctic University of Norway, Tromsø, Norway; Universite Paris Diderot, FRANCE

## Abstract

*N*-acetylneuraminate lyases (NALs) are enzymes that catalyze the reversible cleavage and synthesis of sialic acids. They are therefore commonly used for the production of these high-value sugars. This study presents the recombinant production, together with biochemical and structural data, of the NAL from the psychrophilic bacterium *Aliivibrio salmonicida* LFI1238 (AsNAL). Our characterization shows that AsNAL possesses high activity and stability at alkaline pH. We confirm that these properties allow for the use in a one-pot reaction at alkaline pH for the synthesis of *N*-acetylneuraminic acid (Neu5Ac, the most common sialic acid) from the inexpensive precursor *N*-acetylglucosamine. We also show that the enzyme has a cold active nature with an optimum temperature for Neu5Ac synthesis at 20°C. The equilibrium constant for the reaction was calculated at different temperatures, and the formation of Neu5Ac acid is favored at low temperatures, making the cold active enzyme a well-suited candidate for use in such exothermic reactions. The specific activity is high compared to the homologue from *Escherichia coli* at three tested temperatures, and the enzyme shows a higher catalytic efficiency and turnover number for cleavage at 37°C. Mutational studies reveal that amino acid residue Asn 168 is important for the high *k*_cat_. The crystal structure of AsNAL was solved to 1.65 Å resolution and reveals a compact, tetrameric protein similar to other NAL structures. The data presented provides a framework to guide further optimization of its application in sialic acid production and opens the possibility for further design of the enzyme.

## Introduction

Sialic acids are sugars found on the surface of both prokaryotic- and eukaryotic cells and belong to the family of nine carbon α-keto acidic monosaccharides. *N*-acetylneuraminic acid (Neu5Ac, also often called sialic acid) is the most studied sugar within this family [1–3]. Extensive research has been done after its discovery in 1936 [4, 5] due to its interesting and important biological roles [6–10]. The applications of sialic acid and its derivatives are increasing. They have a wide range of potential medical applications, such as anti-viral and anti-microbial agents [11–14]. Furthermore, Neu5Ac has potential as a glyconutrient and its importance for fetal brain development has made it an attractive component for infant formulas [15]. As a marker, increased concentrations of free serum sialic acid is an indicator of several diseases [16].

The many promising applications of sialic acid have led to an increased interest in developing more efficient methods for production than chemical synthesis, natural product extraction and whole-cell biotransformation can yield [17–23]. In recent years, more efficient and perhaps also environmentally better enzymatic alternatives for large scale production of Neu5Ac have been developed [24]. However, limited documentation of the economic profitability is available.

**Fig 1:**
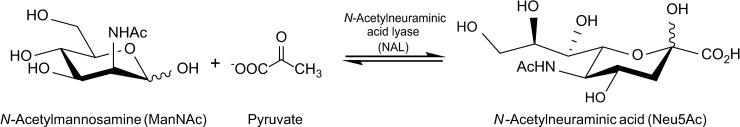
The reversible condensation reaction between ManNAc and pyruvate giving Neu5Ac catalyzed by NAL.

Enzymatic synthesis from *N*-acetylmannosamine (ManNAc) and pyruvate using *N*-acetylneuraminate lyase (NAL, EC 4.1.3.3) as a catalyst is commonly used. NAL is a class I aldolase, and its biological role is to cleave Neu5Ac, however, at favorable conditions, the reverse aldol condensation reaction can be utilized *in vitro* to synthesize Neu5Ac from pyruvate and ManNAc [25] (Fig 1).

For a more optimal economy, Neu5Ac can be produced from the inexpensive *N*-acetylglucosamine (GlcNAc) as starting material [18, 22]. The enzyme GlcNAc 2-epimerase (AGE, EC 5.1.3.8) catalyzes the epimerization of GlcNAc to ManNAc, but it is also possible to use chemical, alkaline epimerization [26]. The rate-limiting step has been shown to be the condensation reaction between ManNAc and puruvate [22]. To push the equilibrium towards synthesis of Neu5Ac, an excess of pyruvate or GlcNAc can be used. Optimization is then achieved by managing the ratios of substrates and enzymes and other reaction parameters [22, 27, 28]. A critical factor for the processes is the enzymes themselves, motivating the characterization of suitable candidates for this application. A NAL with a higher catalytic efficiency would for example increase the speed of the rate-limiting step.

NALs generally tolerate a wide range of acceptor substrates which can be useful for synthesis of Neu5Ac analogues [29]. Characterization and structural studies allow for a further understanding of their requirements and opens for the possibility of their engineering. NALs have also attracted interest as potential drug targets [30], because some pathogenic bacteria can utilize sialic acids as carbon source [31].

NALs have previously been cloned and purified from several mesophilic organisms [30, 32–43], and the X-ray structures are known for *Escherichia coli* (EcNAL) [32, 44–46], *Haemophilus influenza* (HiNAL) [47], *Stapholycoccus aureus* (SaNAL) [30, 40] and *Pasteurella multocida* (PmNAL) [48]. However, biochemical or structural characterization of NALs from psychrophilic (cold adapted) bacteria has so far, to our knowledge, not been reported. Enzymes from psychrophilic organisms are often characterized by having increased catalytic efficiency, a more flexible structure and a lower thermal stability compared to their mesophilic and thermophilic counterparts [49, 50]. These unique properties may prove to be favorable from both a commercial and environmental perspective.

In this paper, we describe the recombinant production, biochemical characterization and structure determination of a *N*-acetylneuraminate lyase from the psychrophilic bacterium *A*. *salmonicida* (AsNAL) [51]. Additionally, we have compared the catalytic properties of AsNAL and two mutants with the commercially available EcNAL. The reported features of the enzyme makes it a promising biocatalyst that may have the potential to provide a more efficient production of sialic acid upon further optimization. The study is part of a larger project where we have targeted several enzymes from local bioprospecting projects, all involved in sialic acid metabolism, and are elucidating the structural and biochemical features of these enzymes.

## Materials and methods

### Bacterial strains and plasmids

*A*. *salmonicida* strain LFI1238 (NCBI Taxonomy ID 316275) was obtained from the Norwegian Institute of Fisheries and Aquaculture Research culture collection, Tromsø, Norway. Genomic DNA was extracted using the Wizard Genomic DNA Purification Kit (Promega, Madison, WI, USA), following the manufacturers’ instructions. Chemically competent Top 10 cells, pDONR221, pDEST14, pDEST17 and *E*. *coli* One Shot BL21 Star DE3 strain were from Invitrogen-Life Technologies (Carlsbad, CA, USA). The genome of the host strain does not contain a gene encoding NAL.

### Cloning and expression

Two constructs of the gene (*nanA*) encoding AsNAL (WP_012549679) were designed and amplified using polymerase chain reaction (PCR). The first construct contains a hexahistidine (His_6_) tag and a Tobacco Etch Virus (TEV) cleavage site in the N-terminus, whereas the second construct contains a His_6_-tag at the C-terminus. The PCR primers were from Sigma-Aldrich (St. Louis, MO, USA) and are shown in Table A in S1 Appendix. Details of the cloning procedure are also described in S1 Appendix. The destination vectors containing the *nanA* constructs were used to transform chemically competent *E*. *coli* TOP 10 cells. The expression plasmids were purified using Plasmid DNA Purification Kit (Qiagen, Hilden, Germany) and sequenced to confirm their identity. *E*. *coli* One Shot BL21 Star DE3 cells were used for large scale expression. A 10 mL overnight preculture (Luria Broth (LB) medium or Terrific Broth (TB) medium containing 100 ug/mL ampicillin) was used to inoculate 1 L of sterile growth-medium. Cells were grown in an orbital shaker at 180 rpm and 37°C until OD_600_ reached 0.6. Protein expression was then induced by adding 0.5 mM isopropyl β-D-1-thiogalactopyranoside (IPTG) after reducing temperature to 20°C. The cells were grown further overnight. The cells were harvested by centrifugation at 9000 x g (JLA 8.1000 rotor) for 25 min at 4°C.

Two single mutants of AsNAL (N168A and N168T) were constructed using the QuickChange II site directed mutagenesis kit from Stratagene (Stratagene, Agilent Technologies Company, USA). The sequence of the primers used for the mutations are listed in Table A in S1 Appendix. The Stratagene protocol was followed with a few modifications. Phusion polymerase was used instead of *PfuUltra* high-fidelity (HF) DNA polymerase. *Dpn* I digestion was performed for 1 h 45 min. *Dpn* I treated DNA (3 μL) were transformed into chemically competent *E*. *coli* TOP 10 cells (Invitrogen). The expression plasmids were purified using Plasmid DNA Purification Kit (Qiagen, Hilden, Germany) and sequenced to confirm their identity. *E*. *coli* One Shot BL21 Star DE3 cells were used for large scale expression. The mutants were expressed following the similar procedure as the AsNAL wild type.

### Purification

Bacterial cell pellets were resuspended in lysis buffer (50 mM Tris-HCl pH 7.5, 500 mM NaCl, 5 mM 2-Mercaptoethanol (β-ME), 10% Glycerol) containing an ethylenediaminetetraacetic acid (EDTA)-free proteinase inhibitor cocktail tablet (Roche Applied Science, Mannheim, Germany) and DNAseI (Invitrogen-Life Technologies, Carlsbad, CA, USA). The cells were disrupted by sonication (Vibra-cell, Sonics & Materials, Newton, CT, USA) on ice using pulse on/off 9.9 s, temperature set to 20°C, amplitude to 25% and total sonication time 30 min. The sonicated extract was centrifuged to remove cell debris (9000 x g, 30 min, 4°C). Purification was carried out at room temperature using Äkta Explorer purification system (GE Healthcare, Uppsala, Sweden). Filtered (0.45 um) crude protein extract (about 40 mL) was loaded onto a HisTrap affinity column equilibrated with buffer A1 (50 mM Tris-HCl pH 7.5, 500 mM NaCl, 10 mM Imidazole, 5 mM β-ME and 10% Glycerol). Loosely bound impurities were washed out with 5% buffer B1 (50 mM Tris-HCl pH 7.5, 500 mM NaCl, 500 mM Imidazole, 5 mM β-ME and 10% Glycerol). Bound protein was eluted using a gradient of 5–100% buffer B1. For the construct with a TEV-cleavable N-terminal His_6_-tag, fractions containing the enzyme were pooled and dialyzed overnight in TEV-cleavage buffer (50 mM Tris-HCl pH 7.5, 500 mM NaCl, 1 mM β-ME and 1 mM EDTA) using Pierce Slide-A-Lyzer dialysis cassettes with a 3.5 kDa molecular weight cutoff, (Thermo Fisher Scientific, Schwerte, Germany) and further digested overnight with TEV protease (1 mg of TEV protease per 5 mg of AsNAL) to remove the His_6_-tag from the protein. After digestion, the mixture was dialyzed again overnight in buffer A1 and loaded onto a HisTrap affinity column equilibrated with buffer A1. The digested protein was collected in the flow-through. The enzyme was concentrated to 5 mL by using a 10 kDa cutoff Amicon Ultra spin-column (Millipore, Billerica, MA, USA) and loaded onto a Superdex 200 prep grade HiLoad (16/60) Gel filtration column equilibrated in buffer A2 (50 mM Tris-HCl pH 7.5, 500 mM NaCl, 5 mM β-ME and 10% Glycerol). The construct with C-terminal His_6_-tag was purified using only one HisTrap step. The purity of the protein was assessed by SDS-PAGE (S1 Fig) using Tris-HCl Mini-PROTEAN TGX Precast gels (Bio-Rad Laboratories, Hercules, CA, USA) and bands of interest were excised from the gel and analyzed by mass spectrometry (Q-TOF UltimaGlobal MS, Micromass, Manchester, UK) to confirm purification of the correct protein. Native molecular weight of the protein was determined by size exclusion chromatography and native PAGE (S1 Fig). Protein concentrations were determined by using both a nanodrop spectrophotometer and the Bio-Rad Protein Assay (Bio-Rad Laboratories, Hercules, CA, USA) [52], according to the microtiter plate protocol described by the manufacturer using bovine serum albumin (BSA) as a standard.

### Enzyme activity assay

Both the condensation and cleavage activities of NAL were assessed using the modified thiobarbituric acid (TBA) assay developed by Aminoff [53] and Warren [54]. Protein used for assays was fash-freezed and stored at -80°C until use (in 50 mM Tris-HCl pH 7.5 and 250 mM NaCl). The condensation activity was determined by incubating 50 μL of a reaction mixture containing 15 mM sodium pyruvate, 15 mM ManNAc, 125 mM HEPES pH 8.0 and different concentrations of enzyme depending on assay type. Concentrations of different reaction components were adjusted according to Suryanti et al. [55]. The reaction was terminated by adding 137 μL 2.5 mg/mL sodium periodate in 57 mM H_2_SO_4_, followed by incubation at 37°C for 15 min with shaking at 1350 rpm. Sodium arsenite (50 μL, 25 mg/mL sodium arsenite in 0.5 M HCl) was added resulting in brown color. The tubes were shaken manually until the brown color disappeared. 2-thiobarbituric acid solution (100 μL, 71 mg/mL adjusted to pH 9.0) was subsequently added, and the tubes were incubated in boiling water for 7.5 min, then on ice for 5 min and at room temperature for 5 min. The red chromophore was extracted by addition of acidic butanol (1 mL of butanol containing 5% HCl) and horizontal shaking for 10 min. Tubes were centrifuged at 16000 x g, 7 min (room temperature) to separate the organic and inorganic phases. The organic phase containing the red chromophore (200 μL) was used for measurement of absorbance at 549 nm in a spectrophotometer (SpectraMax M_2_^e^, Molecular Devices, Sunnyvale, CA, USA). The amount of Neu5Ac produced was inferred from a standard curve. To generate a standard curve, different concentrations of Neu5Ac (0.031–1 mM) were treated with 137 μL of 2.5 mg/mL sodium periodate in 57 mM H_2_SO_4_ and the TBA assay procedure was followed as described above.

The cleavage activity was determined by incubating 50 μL of a reaction mixture containing 5 mM Neu5Ac, 125 mM HEPES pH 8.0 and different concentrations of enzyme.Termination of the reactions and subsequent steps of the assay are as described above. The decrease in absorbance is correlated to the increase in cleavage activity. All experiments were performed in triplicate.

### Activity at different pH values and temperatures

pH profiles were determined by assaying the enzyme in triplicate for both the condensation and the cleavage directions at pH values ranging from 5.5 to 11.0 (buffers used are described in S2 Fig). The reaction mixture was incubated at room temperature for 1 h before being subjected to the TBA assay. Temperature profiles were determined for both directions by assaying the enzyme in triplicate from 4 to 80°C in HEPES pH 8.0. The reaction mixture was incubated for 30 min at selected temperatures and the reaction was terminated by adding 2 μL of concentrated H_2_SO_4_, and then subjected to the TBA assay.

### Condensation-cleavage equilibrium studies

In order to determine the equilibrium constant between reactants and products, activity at different temperatures was determined by incubating 50 μL of reaction mixtures containing enzyme, 125 mM HEPES pH 8.0, either 5 mM Neu5Ac (cleavage) or 5 mM ManNAc and 5 mM pyruvate (condensation) at 4, 23 and 37°C. Aliquots of samples were taken out at selected intervals and the reaction stopped by adding 137 μL 2.5 mg/mL sodium periodate in 57 mM H_2_SO_4_ and further processed according to the TBA assay. The reaction was followed until there was no further change in absorbance, and hence, the reaction had reached equilibrium. Equilibrium concentrations of Neu5Ac at 4, 23 and 37°C were determined by calculating the average of uncleaved Neu5Ac (cleavage reaction) and synthesized Neu5Ac (condensation reaction) at the equilibrium, because both reactions converge towards this equilibrium value. The equilibrium concentrations were used to calculate the equilibrium constants (*K*_c_) for the condensation direction at the respective temperatures by the formula $K_{c}=\frac{\left[Neu5Ac\right]}{\left[ManNAc][pyruvate\right]}$. A Van’t Hoff plot of 1/T versus ln*K*_c_ of own values and literature values were used to calculate change in enthalpy (ΔH) and change in entropy (ΔS). Using linear regression, the equation for the line on the form *y* = *ax*+*b* was found. The Van’t Hoff Equation is known as $\ln K_{c}=-\frac{\Delta H}{RT}+\frac{\Delta S}{R}$, and has a slope of −ΔH/*R* and an intercept equal to ΔS/*R*. Calculations were performed using the gas constant *R* of 1.987x10^-3^ kcal K^-1^ mol^-1^. The change in free energy (ΔG) was calculated using the relationship ΔG = ΔH − TΔS.

### Effect of substrate ratio and temperature shift on production yield of Neu5Ac

The effect of substrate ratio on the conversion yield of Neu5Ac was studied by varying the pyruvate concentration while the concentration of ManNAc was kept constant. The pyruvate concentration ranged from 2.5 to 70 mM, whereas the ManNAc concentration was 5 mM, resulting in a pyruvate:ManNAc ratio ranging from 0.5 to 14. The condensation reaction mixtures additionally contained 125 mM HEPES pH 8.0 and enzyme, and were incubated at room temperature for 7.5 h.

The aldol condensation is an exothermic reaction; hence, lowering the temperature should increase the yield of Neu5Ac. Thus, a temperature shift experiment was carried out to see how much the Neu5Ac production could be increased by altering the equilibrium once it had been achieved. The reaction mixture (enzyme, 50 mM pyruvate, 5 mM ManNAc, 125 mM HEPES pH 8.0) was incubated at room temperature (23°C) for 7.5 h and after reaching equilibrium it was shifted to 4°C and incubated for 15 h. As a control, one reaction was kept at room temperature and another at 4°C for all the time. The difference in yield between the temperature shifted and non-shifted reactions were calculated. The standard TBA assay was used to assess the activity.

### Stability of AsNAL

Long term stability of AsNAL at different pH values was studied by incubating the enzyme at pH 6.0 to 11.0 at room temperature for a month (S4A Fig). The decrease in activity was calculated compared to initial activity at the respective pH values. The enzyme activity in the condensation direction was measured using the standard reaction mixture incubated at room temperature for 1 h, with subsequent TBA assay. The pH stability was also studied using the thermofluor method [56]. For the thermofluor assay, the protein was dialyzed overnight at 4°C against a buffer containing 10 mM HEPES pH 7.5, 150 mM NaCl and 2 mM β-ME. The dialyzed protein was mixed with 2 μL of 300x Sypro Orange protein gel stain (Sigma-Aldrich, St. Louis, MO, USA) and 100 mM of different buffers ranging from pH 5.0 to pH 9.0 to a final volume of 25 μL (S4B Fig). Thermal shifts were screened for by heating in an iCycler iQ Real Time PCR Detection System (Bio-Rad Laboratories, Hercules, CA, USA) from 1 to 80°C in increments of 1°C/min.

The melting temperature of AsNAL was studied by differential scanning calorimetry (DSC) using a Nano Differential Scanning Calorimeter III (Calorimetry Sciences Corporation, MA, USA). Protein was dialyzed overnight at 4°C against 50 mM HEPES pH 7.5 and 500 mM NaCl, filtered and then degassed for 15 min and concentrated to 1.9 mg/ml. Thermal denaturation was followed between 1 to 80°C using a heating/cooloing rate of 1°C/min and the dialysis buffer was used as reference buffer in the runs. The NanoAnalyze software was used to calculate the melting temperature by the substraction of the buffer-buffer baseline from the protein scan and fitting the data toa two-state transition model. The results are presented in the supporting information (S5 Fig).

### Comparative studies of specific activity and determination of kinetic constants

The specific activity of AsNAL, in both directions, was compared to the specific activity of the commercially available EcNAL (Sigma) at three different temperatures: 4, 23 and 37°C. Standard reaction mixtures were incubated at room temperature for 1 h before being subjected to the TBA assay.

The enzyme kinetics for the cleavage reactions for AsNAL, EcNAL and the AsNAL mutants N168A and N168T were studied using a lactate dehydrogenase (LDH)-coupled continuous assay [35, 57, 58]. The incubation mixtures contained variable amounts (1, 5, 15, 30, 45, 60, 75 and 90 mM) of Neu5Ac, 50 mM Tris-HCl pH 8.5, 0.15 mM NADH (Sigma Aldrich, St. Louis, MO, USA) and 4 U LDH (Sigma Aldrich, St. Louis, MO, USA). The amount of enzyme used were 0.56, 2.3, 8.5 and 7.9 μg for AsNAL, EcNAL, AsNAL N168A and AsNAL N168T, respectively. Assay volumes were 200 μl. Components, except the enzyme, were mixed and incubated at 37°C for 5 min before the reactions were started by adding enzyme. The measurements were performed in triplicates. The decrease in absorbance at 340 nm, corresponding to the oxidation of NADH by LDH in presence of released pyruvate, was measured spectrophotometrically using a Spectramax M_2_^e^ Microplate reader. Initial velocities were calculated using the SoftMax Pro software and subsequently fitted to the Michaelis-Menten equation using the program GraphPad Prism 5 (GraphPad Software Inc., CA, USA). The turnover number (*k*_cat_) were calculated using the formula *V*_max_/[Enzyme], where *V*_max_ is the maximum velocity. The relationship between absorbance and substrate concentration was calculated from a standard-curve obtained by measuring the maximum absorbance from various substrate concentrations. The relationship is given by the formula: *y* = 0.0031*x* + 0.0052, where x is the pyruvate concentration. By using this formula, values of *V*_max_ were converted from mOD/min to μM/min. Enzyme concentrations were converted from mg/mL to molar using the calculated molecular mass of 32257.9 g/mol (monomeric protein).

### Use of AsNAL in a one-pot reaction with *N*-acetylglucosamine (GlcNAc) and pyruvate at alkaline pH

20 mM of GlcNAc or ManNAc, 80 mM of pyruvate, 7 μg of enzyme (AsNAL or EcNAL), 125 mM buffer and dH_2_0 were mixed in a tube to a total volume of 250 μL. For ManNAc reactions, the buffer used was HEPES pH 8.0. Initially, different buffers were tested for GlcNAc reactions. For further experiments, the optimal buffer, CAPS pH 11.0 was used. Experiments were performed in triplicate. For the ManNAc experiments, aliquots were sampled after 0.5, 1.0, 1.5, 2.5 and 4.0 h. For the GlcNAc experiments, aliquots were sampled after 12, 24, 36, 48 and 72 h. Reactions were terminated by addition of 2 μL concentrated H_2_SO_4_. The TBA assay was followed to determine the amount of Neu5Ac produced. The activity was corrected for a blank value without enzyme. The experiments with ManNAc and the optimal buffer from the pH activity experiments (HEPES pH 8.0) were comparative experiments.

### Crystallization, data collection, structure determination and analysis

Protein used for crystallization was stored in purification buffer of pH 7.5 and concentrated to 9.6 mg/ml prior to crystallization. Initial crystallization trials were set up using an Art Robbins Phoenix crystallization robot to create 96-well crystallization setups using 60 μL in the reservoirs, and 200 nL protein solution plus 200 nL reservoir solution in the experimental drops. Both commercial and homemade stochastic screens were tried. Promising conditions were subsequently optimized in 48-well hanging drop plates using reservoirs of 500 μL and 1 μL plus 1 μL drops.

X-ray data on AsNAL crystals were collected at BL14.1 at Bessy. Data were processed in XDS [59] and SCALA and TRUNCATE of the CCP4 program suite [60]. Despite low sequence identity to known homologous structures, the structure of AsNAL could be solved by molecular replacement using the auto-rickshaw server (http://www.embl-hamburg.de/Auto-Rickshaw/) [61], suggesting PDB entry 1F74 [47] as search model. Automatic re-tracing of the polypeptide chain was carried out with ARP/wARP [62]. Subsequent improvement of the model was made by alternate cycles of manual refitting of amino acids using Coot [63] based on sigma-weighted 2mFo-DFc and mFo-DFc electron density maps and refinement using Refmac5 [64] of the CCP4 suite. Programs for structural comparison and analysis included the CCP4 suite, the DaliLite server (http://ekhidna.biocenter.helsinki.fi/dali_lite/) [65], the PISA server ‘Protein interfaces, surfaces and assemblies’ service at the European Bioinformatics Institute (http://www.ebi.ac.uk/pdbe/prot_int/pistart.html) [66] and the Protein structure comparison service Fold at the European Bioinformatics Institute (http://www.ebi.ac.uk/msd-srv/ssm) [67].

Illustrations of the 3D structure were made in PyMOL (DeLano Scientific; http://www.pymol.org) and electrostatic surface potentials were generated using the APBS plugin (Adaptive Poisson-Boltzmann Solver) [68]. The structure based sequence alignment was generated using the ESPript server (http://espript.ibcp.fr/ESPript/ESPript/) [69]. The structure has been deposited in the Protein Data Bank, entry 5AFD.

## Results

### Expression, purification and effect of His_6_-tag on the enzymatic properties

AsNAL was soluble when expressed at 20°C and pure protein was obtained for both constructs. The protein was initially purified with a His_6_-tag present either in the N-terminal (cleavable, shown in S1 Fig) or the C-terminal (uncleavable) of the protein. The enzyme yield after His_6_-tag purification was 50 mg/L using LB medium and 450 mg/L in TB medium. The protein with the N-terminal His_6_-tag removed was used for the kinetics and the comparative studies of specific activity. For other characterization experiments, the protein with the N-terminal His_6_-tag present was used, but it did not affect the activity (not shown). The activity was not affected by presence of the C-terminal His_6_-tag either, which was used for the structural studies. The purity of the protein after size exclusion chromatography, in addition to native PAGE analysis indicating the tetrameric entity, is shown in the supporting information (S1 Fig).

### pH and temperature profiles

The effect of pH on the enzyme activity was studied for both the condensation and cleavage reactions and is shown in the supporting information (S2A and S2B Fig). The enzyme was active over a wide pH range for both reactions, with maximum activities between pH 7.5–8.5 (S2A and S2B Fig). The temperature profiles are shown in S2C Fig. The optimal temperature was 20°C for the condensation reaction and 65°C for the cleavage reaction.

### Equilibrium reaction studies and effect of substrate ratio and temperature shift on conversion yield

**Fig 2:**
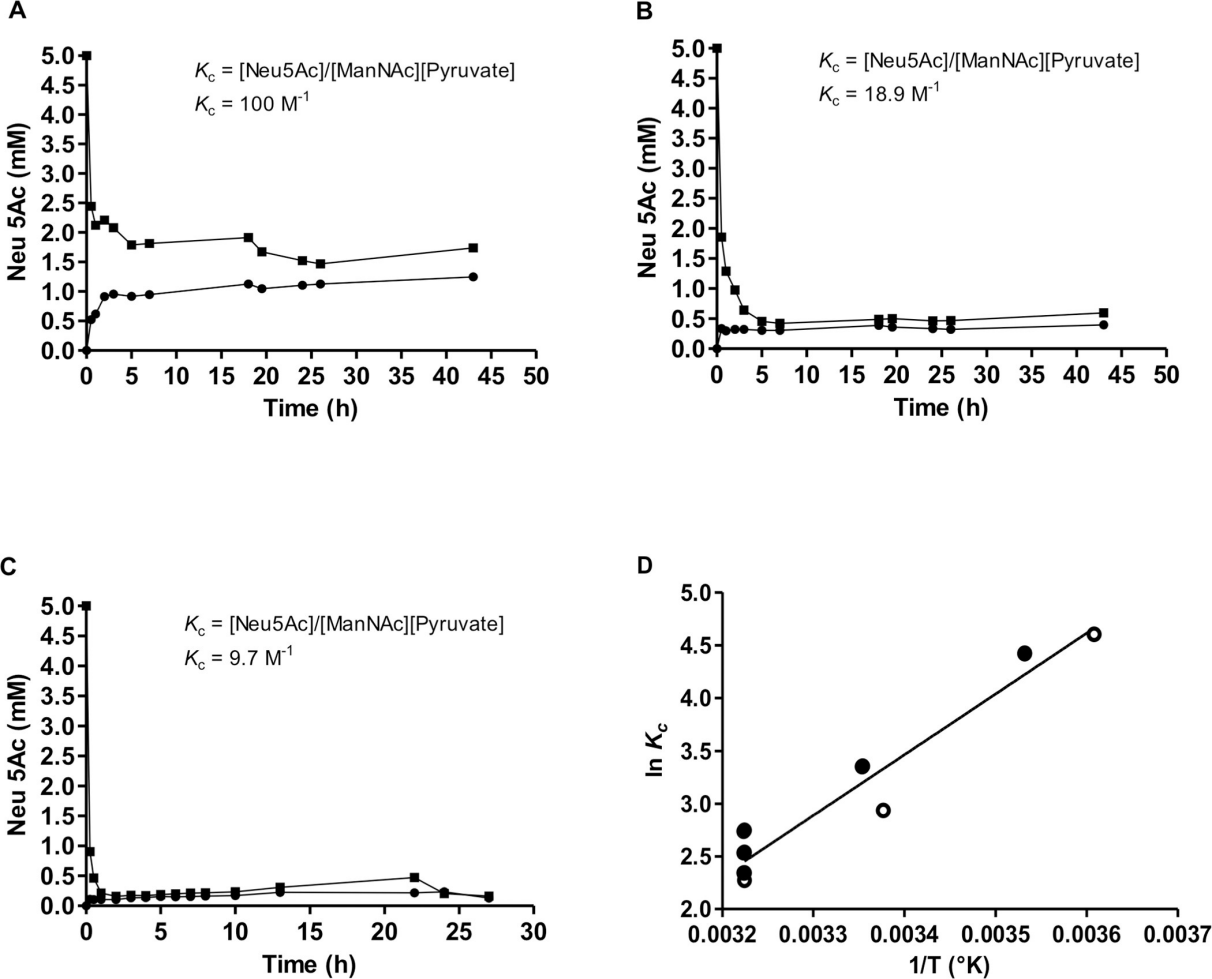
Equilibrium plots of AsNAL condensation and cleavage ractions and Van’t Hoff plot of 1/T versus ln*K*_c_. Equilibrium plots of the AsNAL condensation reaction (black circles), showing the amount of Neu5Ac being produced, and the cleavage reaction (black squares) showing the uncleaved amount of Neu5Ac at different temperatures. (A) Reactions at 4°C, (B) Reactions at 23°C, (C) Reactions at 37°C. (D) A Van’t Hoff plot of 1/T versus ln*K*_c_ where open circles indicate ln*K*_c_ values from our experiment at 37, 23 and 4°C (shown from left to right) and closed circles indicate literature ln*K*_c_ values at 37, 25 and 10°C (from left to right).

The equilibrium between reactants and products in the reactions catalyzed by AsNAL were studied at three different temperatures; 4, 23 and 37°C (Fig 2A–2C). The equilibrium concentration of Neu5Ac at 4, 23 and 37°C were 1.3, 0.4 and 0.2 mM respectively. The apparent equilibrium constant for the condensation reaction, *K*_c_, was calculated to be 100.0 M^-1^ at 4°C, 18.9 M^-1^ at 23°C and 9.7 M^-1^ at 37°C. The Van’t Hoff plot of 1/T versus ln*K*_c_ is shown in Fig 2D. Here, we also included other literature values for *K*_c_ [27, 33, 57, 70]. Performing a linear regression, we found the function for the relationship to be y = 5753.2x-16.097, which was used to determine change in entalphy (ΔH) and change in entropy (ΔS). The equilibrium constants and calculated thermodynamic parameters for the reaction at different temperatures are given in the supporting information (S1 Table).

The effect of substrate ratio showed a gradual increase in Neu5Ac production with increasing pyruvate concentration while keeping the ManNAc concentration constant. The highest yield was observed with the ratio of 14:1 (pyruvate 70 mM: ManNAc 5 mM, S3A Fig). Shifting the reaction temperature from 23°C to 4°C increased the yield of Neu5Ac by 30% (S3B Fig).

### AsNAL stability

The pH-stability of AsNAL in various buffers is shown in the supporting information (S4 Fig). The protein appears to be relatively stable at higher pH as interpreted from the lower decrease in activity for the condensation reaction. More than 83% of the activity was retained at all measured pH values after one month (S4A Fig).

Thermal denaturation of AsNAL using the thermofluor-method was used to study the stability of the protein at different pH values. In milli-Q water, the melting temperature of the enzyme (kept in 10 mM HEPES pH 7.5) was 73.1 ± 0.2°C. As a general trend, a low pH buffer solution decreased the melting temperature, whereas higher pH increased it compared to the reference (S4B Fig). The melting temperature for AsNAL determined by DSC was 77.5°C and is shown in the supporting information (S5 Fig).

### Comparative studies of specific activity and kinetic constants belonging to AsNAL, EcNAL and AsNAL mutants

**Fig 3:**
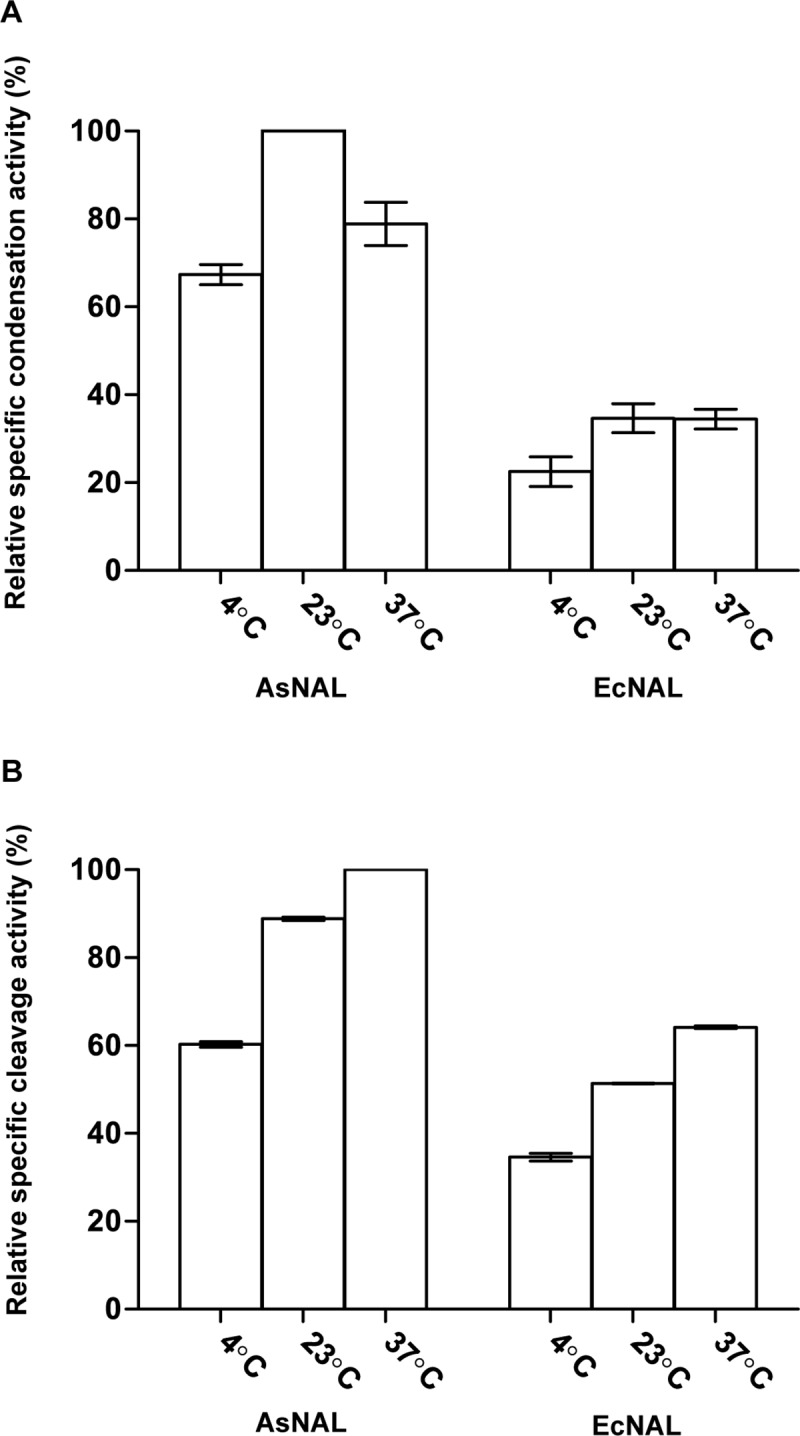
Specific activity of AsNAL and EcNAL at different temperatures. (A) condensation reaction and (B) cleavage reaction.

The specific activity of the condensation reaction ranged between 40–60% higher for AsNAL compared to EcNAL at the tested temperatures (Fig 3A). For the cleavage reaction, the specific activity for AsNAL was 25–35% higher than for EcNAL (Fig 3B).

**Fig 4:**
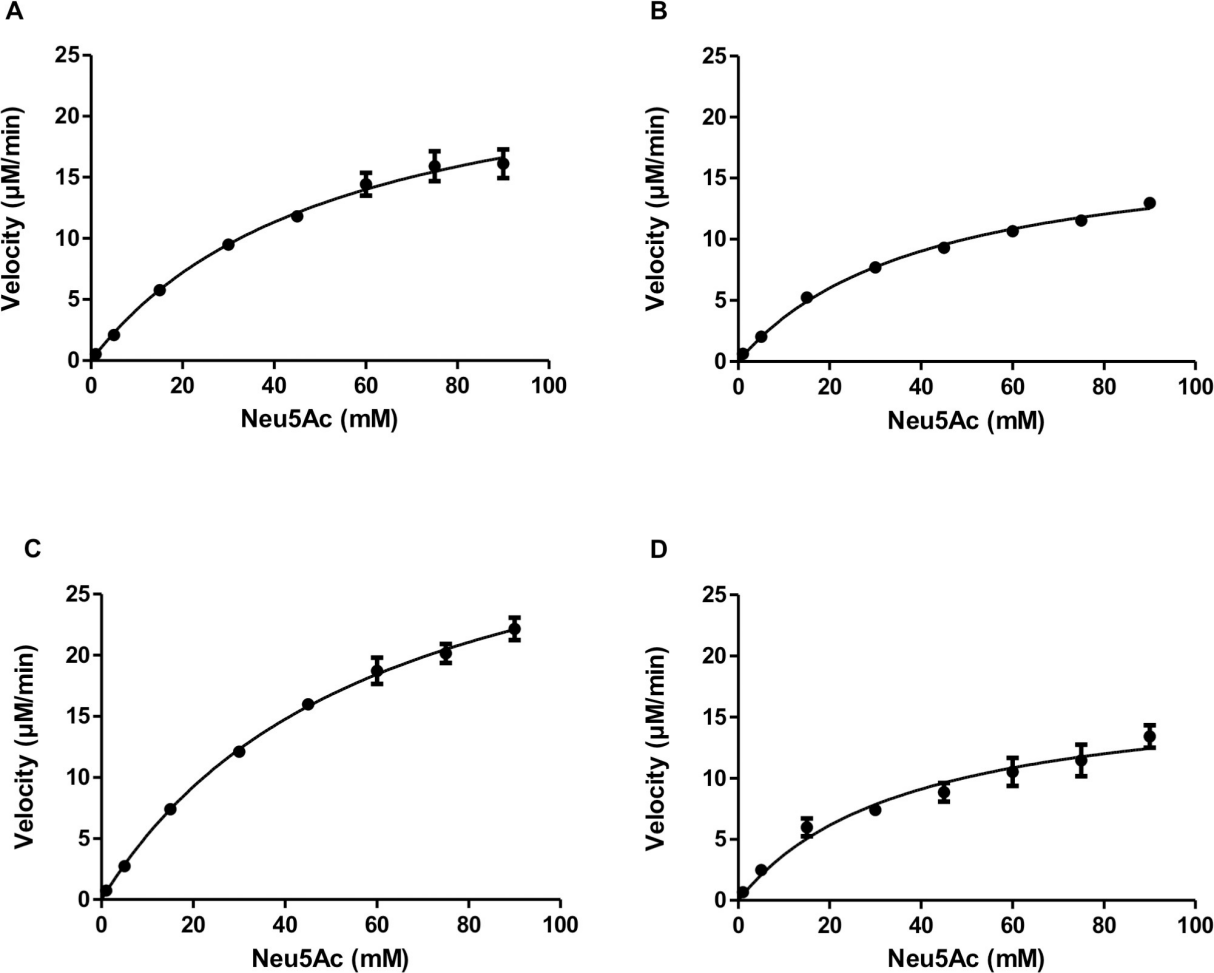
Michaelis-Menten curves for cleavage reactions of Neu5Ac. (A) AsNAL (B) AsNAL N168A (C) AsNAL N168T and (D) EcNAL. Initial velocities at different substrate concentrations were fitted to the Michaelis-Menten equation.

The *K*_M_ (Michaelis constant), *k*_*cat*_ and the catalytic efficiency for the cleavage reaction of AsNAL were compared with the values from the commercially available homologue EcNAL, and are presented in Table 1 and Fig 4A and 4B. The *K*_M_ for AsNAL is 1.4 times higher than the *K*_M_ obtained for EcNAL and the *k*_cat_ is six times higher, and thus the catalytic efficiency is four times higher. To facilitate the comparison, we have summarized the *K*_M_ values and pH-and temperature optima belonging to NALs from different organisms in the supporting information (S2 Table). For AsNAL, the role of residue Asn168 in catalysis was investigated by site-specific mutagenesis. Kinetic constants for the mutants N168T and N168A are shown in Table 1 and Fig 4C and 4D. The most striking difference was that both mutants were found to have *k*_cat_ values less than 10% of the native enzyme.

**Table 1:** Kinetic data measured for the cleavage reaction at 37°C for AsNAL, AsNAL mutants and EcNAL.

Source	Substrate	*V*_max_ (μM/min)	*K*_M_ (mM)	*k*_cat_ (s^-1^)	*k*_cat_*/K*_M_ (M^-1^ s^-1^)
AsNAL	Neu5Ac	26.6 ± 1.6	53.9 ± 6.6	5.12 ± 0.30	95.0
N168A	Neu5Ac	18.1 ± 0.5	40.4 ± 2.7	0.23 ± 0.01	5.7
N168T	Neu5Ac	36.7 ± 1.4	59.5 ± 4.5	0.50 ± 0.02	8.4
EcNAL	Neu5Ac	17.6 ± 1.4	37.3 ± 7.3	0.87 ± 0.07	23.3

### Use of AsNAL in a one-pot reaction with GlcNAc and pyruvate at alkaline pH

**Fig 5:**
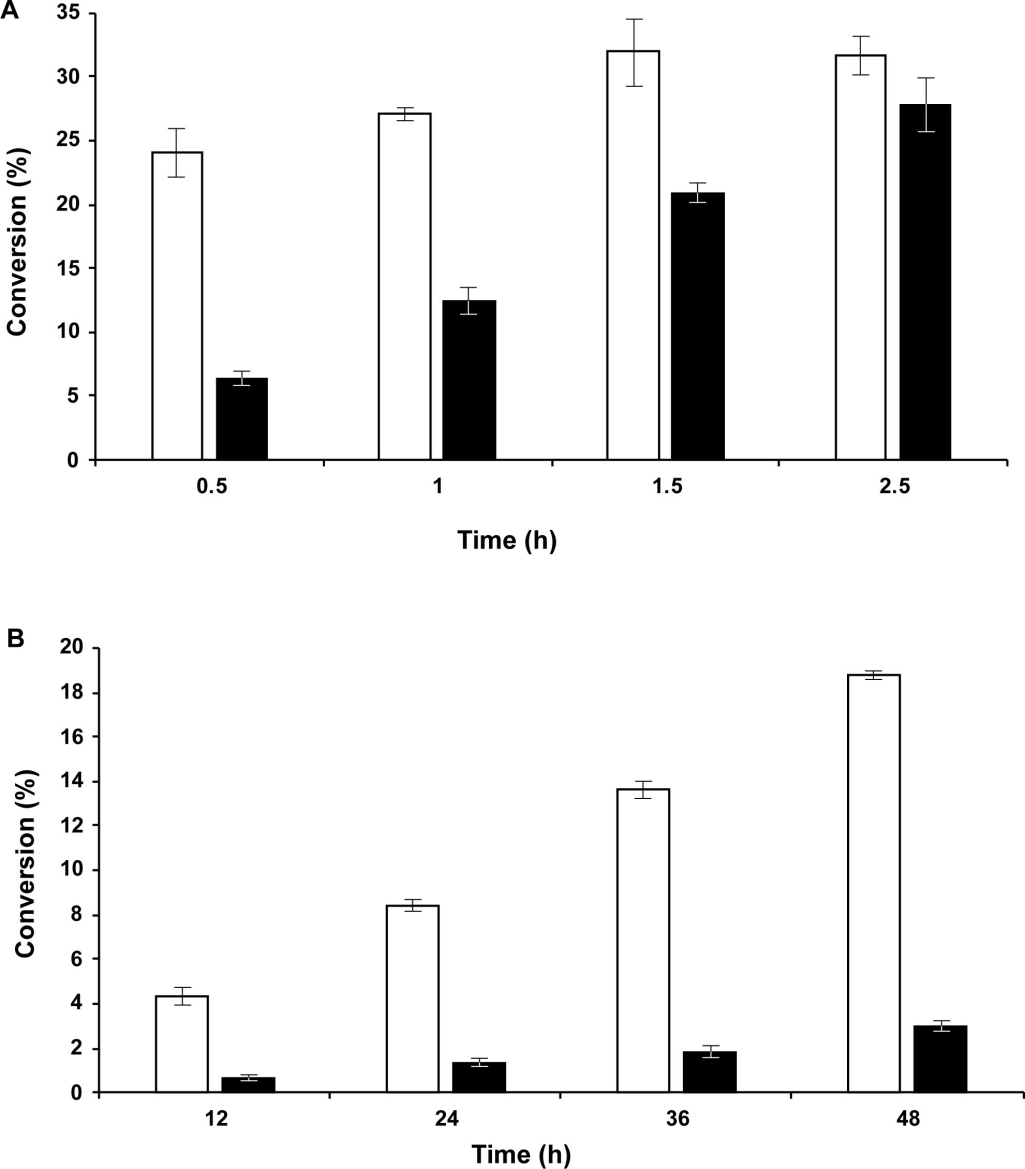
Production of Neu5Ac by AsNAL and EcNAL. (A) by incubation of 7 μg of the enzymes with 80 mM pyruvate and 20 mM ManNAc in 125 mM of HEPES pH 8.0 and (B) by incubation of 7 μg of the enzymes with 80 mM pyruvate and 20 mM GlcNAc in 125 mM of CAPS pH 11.0. AsNAL is shown with withe bars and EcNAL with black bars.

GlcNAc epimerizes chemically to ManNAc at pH values above 9.0, and the epimerization rate increases with increasing pH above this value [26]. We have shown that AsNAL can use the produced ManNAc for the production of Neu5Ac in a one-pot reaction (Fig 5), where the production of Neu5Ac through the NAL condensation reaction is coupled with the alkaline epimerization of GlcNAc to ManNAc. Yields have been compared to the production of Neu5Ac when using pyruvate and ManNAc in 4:1 ratios at pH 8.0, where the production was highest after 1.5 h of incubation with AsNAL (Fig 5A). From Fig 5A it can be seen that AsNAL is more efficient compared to EcNAL, where the difference in production of Neu5Ac is highest within the first hour of incubation. The production of Neu5Ac by EcNAL increases gradually up to 2.5 h, but still converts 14% less than AsNAL. After 4 h of incubation the reactions were completed. The production of Neu5Ac using pyruvate and GlcNAc in a 4:1 ratio at pH 11.0 was compared to the previous experiment and is shown in Fig 5B. The conversion is below 3% for EcNAL at this pH value. For AsNAL, the conversion increases gradually from 4% after 12 h and up to 19% after 48 h. When compared to the highest yield obtained for the ManNAc experiment, the production of Neu5Ac is around 60% for AsNAL and around 10% for EcNAL after 48 h. After 72 h incubation the production flattens out also for AsNAL (not shown) due to a completed reaction.

### Crystallization, data collection and processing

A crystallization condition, composed of 25% PEG 1.5 K and 20% glycerol, produced diamond shaped crystals. The crystals diffracted up to 1.65 Å and belong to the orthorhombic space group *I222* with cell parameters of 67 x 86 x 118 Å^3^ (Table 2). Data collection and refinement statistics is listed in Table 2.

**Table 2:** Data collection and refinement statistics. Outer shell (1.63–1.55 Å) parameters are given in parentheses.

Data collection	
Beam line	Bessy BL14.1
Diffraction limit	1.65
Space group	*I222*
Unit cell parameters	
a-axis (Å)	66.94
b-axis (Å)	86.28
c-axis (Å)	117.73
Total no. of reflections	170659 (24738)
No. of unique reflections	41377 (6000)
Completeness (%)	100.0 (100.0)
I/σ(I)	14.1 (1.5)
Mean I/σ(I)	20.2 (2.8)
R_merge_ (%)	4.3 (50.8)
Multiplicity	4.1 (4.1)
Wilson B (Å^2^)	19.7
**Refinement**	
R_work_ (%)	16.43
R_free_ (%)	20.12
Average *B* factors (Å^2^)	21.27
No. protein atoms	2290
Solvent	187
Glycerol	1
PEG	3
R.m.s. deviations	
Bond lengths (Å)	0.024
Bond angles (°)	2.196
% residues in regions of the Ramachandran plot	
Most favored	89.7
Additionally allowed	10.3
Generously allowed	0
Disallowed	0

**Fig 6:**
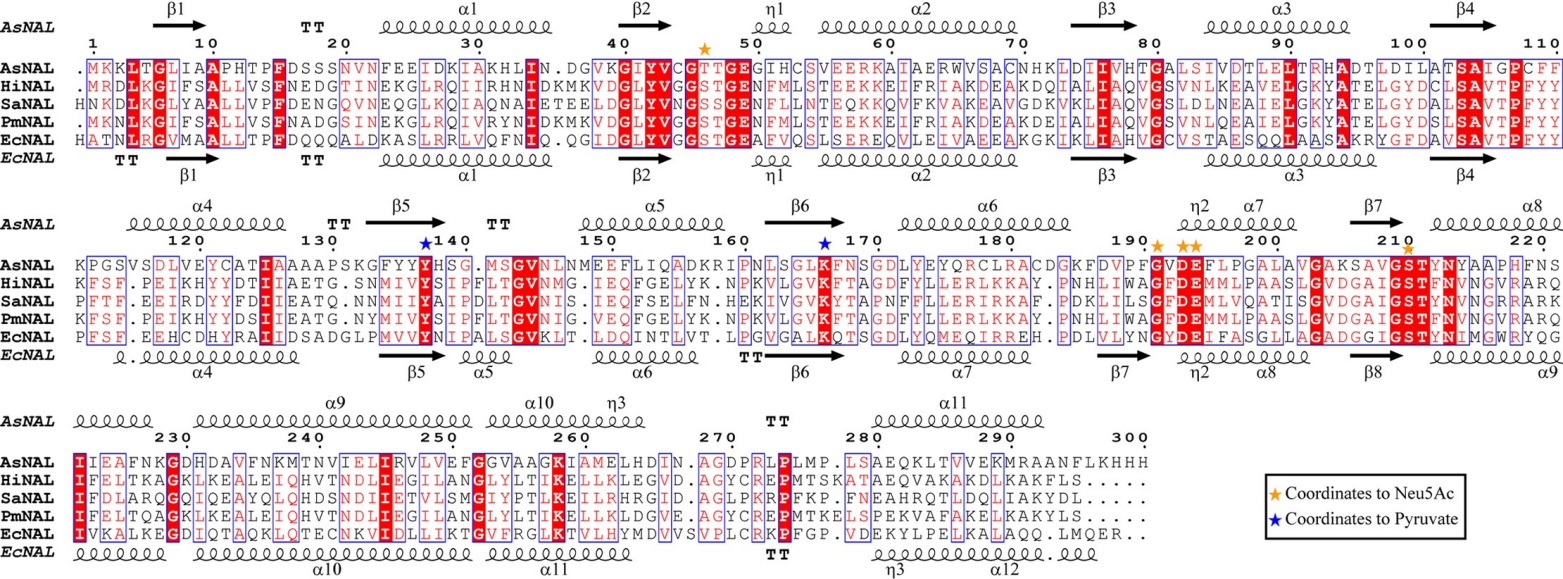
Structure based sequence alignment of different bacterial NALs. Sequences (with accession number in parenthesis) are from *A*. *salmonicida* LFI1238 (5AFD_A); *H*. *influenza* (1F7B_A), *S*. *aureus* (4AH7_A), *P*. *multocida* (4IMD_A); and *E*. *coli* (2WNN_A*)*. The secondary structure elements belonging to *A*. *salmonicida* and *E*. *coli* are indicated above and below the alignment respectively. Residues coordinating to Neu5Ac or pyruvate are indicated by the colored symbols as shown in the box.

**Fig 7:**
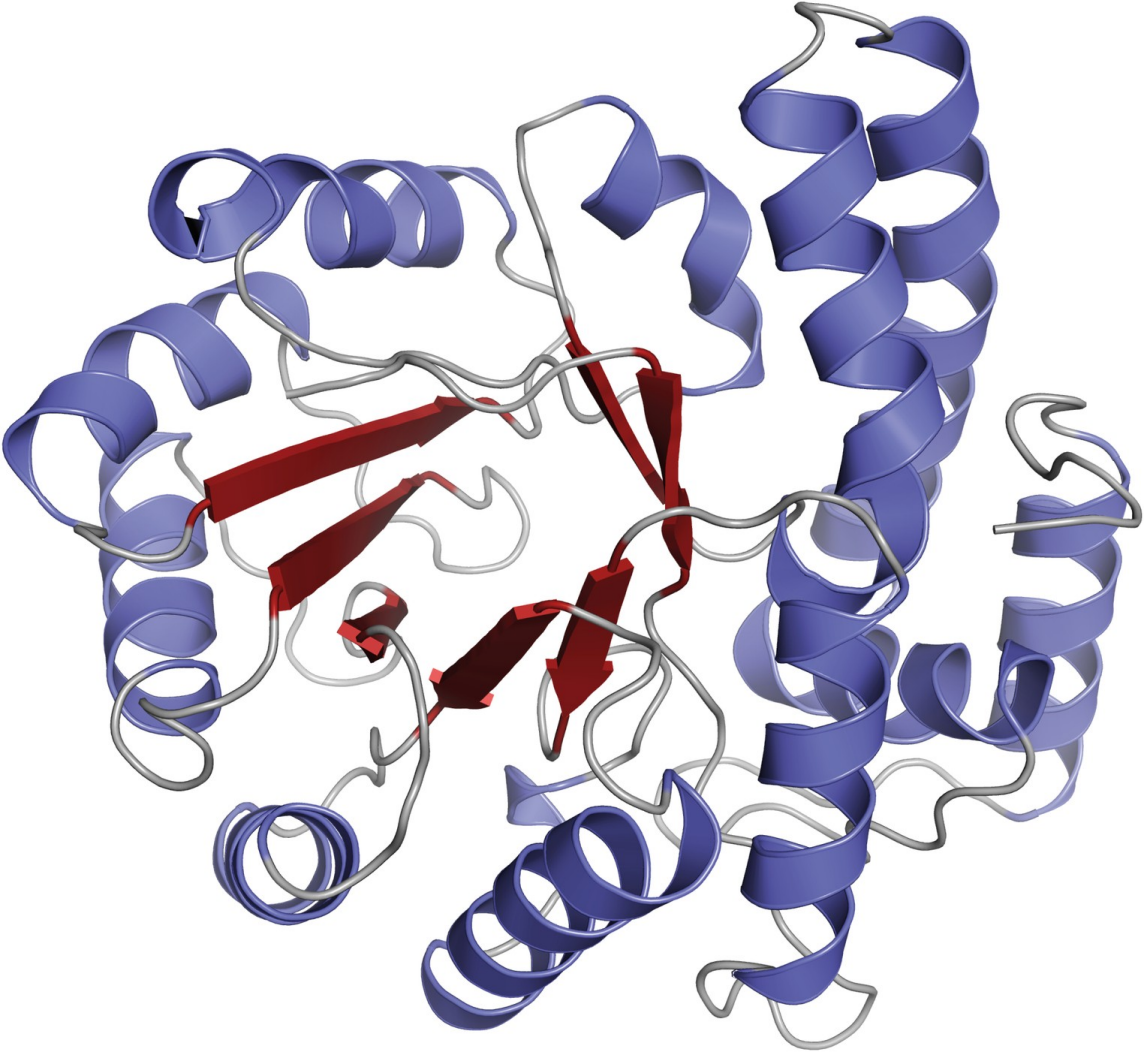
Cartoon representation of one monomer of AsNAL. The cartoon is showing the TIM barrel fold in the center surrounded by helices.

The whole amino acid sequence from residue 1 to 297 could be traced in the electron density maps. Three of the His residues from the tag were also visible in the electron density maps. The three His residues do not appear to have interactions with the rest of the molecule which could have implications for the overall fold. Electron density clearly larger than water molecules was interpreted as glycerol and ethylene glycol originating from the crystallization conditions. There is one molecule in the asymmetric unit, but the crystallographic symmetry generates the functional tetrameric quaternary structure. The active sites are pointing inwards toward the center of a donut shaped tetramer. Despite having less than about 25% sequence identity (Fig 6) to the homologous structures of EcNAL [45], HiNAL [47], SaNAL [40] and PmNAL [48], AsNAL shares similarity in the overall fold, being a TIM barrel consisting of a barrel of 7 parallel strands (the eighth strand is distorted) surrounded by 11 helices (Fig 7). The fold of the core β-barrel is well conserved among AsNAL, EcNAL, HiNAL, SaNAL and PmNAL, but there are small distortions in the orientations of the alpha helices, resulting in an overall rmsd of almost 1.7 Å (according to Protein structure comparison service Fold at European Bioinformatics Institute; http://www.ebi.ac.uk/msd-srv/ssm; [67]). Although the sequence identity between AsNAL and the other NALs is low, residues involved in catalysis and substrate binding are well conserved (Fig 6).

## Discussion

### Purification and oligomeric state of AsNAL

AsNAL could be expressed and purified to homogeneity. The amount of protein obtained after HisTrap purification in LB medium was 50 mg/L and in TB medium it was 450 mg/L. The yield using TB medium is higher than that reported for LpNAL [38] and ScNAL [39], where constant oxygenation were used. From native PAGE and size exclusion chromatography, it is evident that AsNAL is tetrameric in solution. This is in accordance with what is reported for other NALs, [38, 39, 44] although there are reports of EcNAL being a trimer [32, 33] and CpNAL a dimer [35, 71].

### Effect of pH and temperature on activity and stability

AsNAL is active over a wide range from pH values 5.5 to 11.0, and the optimum pH is similar to that of EcNAL [33, 72]. At acidic (5.5) and basic (11.0) pH, AsNAL maintains 60% activity in both directions, whereas the activity of EcNAL has been reported to be lower than 50% [33] (S2A and S2B Fig). The high activity at alkaline pH can be a useful property for industrial applications. We have shown that it is possible to couple the AsNAL condensation reaction in a one-pot reaction at alkaline pH with chemical epimerization of GlcNAc to ManNAc. We have also shown that AsNAL was better suited for this than EcNAL (Fig 5A and 5B). However, the conditions tested are not optimal for the process, and it is worthy of note that higher substrate concentrations will give higher yields.

Both the long term stability study (S4A Fig) and the thermofluor study (S4B Fig) indicated higher stability of AsNAL at higher pH values (S4B Fig). In comparison with other NALs, ScNAL [39] and EcNAL [33] has been reported to be less stable at basic pH. LpNAL showed 60% remaining activity after 15 days of incubation at pH 11.0 [38] which is similar to AsNAL. But after incubation at pH 6.0, the remaining activity for LpNAL was less than 10% which is very low compared to AsNAL at the same pH (S4A Fig).

The temperature optimum for the AsNAL cleavage reaction (65°C) is less than, or similar to, the reported values for other NALs (S2 Table). The optimal temperature for the condensation reaction is 20°C. This is very low compared to any of the reported values to date (S2 Table). The amount of Neu5Ac produced or cleaved is determined by assay conditions such as incubation time, type of buffer and concentration of substrate and enzyme. For example, the incubation time varies slightly for different characterized NALs [32, 33, 41–43, 73]. Thus, the differences in assay setup is a contributing factor for the variations in optimum temperatures for the different NALs. If a longer incubation time had been used in our studies, the optimum temperature for Neu5Ac synthesis would have been lower. This is due to the time it takes to reach equilibrium, and that the equilibrium constant favours the exothermic synthesis reaction when lowering the temperature. In the case of AsNAL, 75% and 30% activity are retained at 10°C for the condensation and cleavage directions, respectively, under the conditions used. This is striking compared to what is observed for other NALs.

The DSC study of AsNAL (S5 Fig) shows that the protein unfolds with a single sharp peak indicating that the tetramer and each monomer unfold simultaneously. The melting temperature coincides well with the result from the thermofluor experiment. These values are quite high for an enzyme from a psychrophilic specie. However, this is probably linked to the tetrameric and compact form of AsNAL which probably contribute to the high thermal stability of the NAL enzymes, as pointed out by Schauer et al. [74].

### Equilibrium constant and effect of substrate ratio and temperature shift on production yield

The equilibrium compositions are affected by various factors such as pH, volume, pressure and temperature. In our study, we obtained two different optimum temperatures for the condensation and the cleavage reactions of AsNAL. This can be explained by a less favourable equilibrium constant at higher temperatures for the condensation reaction [75], which affects the kinetic constants [28]. The *K*_c_ increased with increasing temperature for the endothermic cleavage reaction (Fig 2A–2C). An increase in temperature moves the equilibrium towards the product side meaning that the cleavage of Neu5Ac is more favored at higher temperature. From the Van’t Hoffs plot for the condensation reaction (Fig 2D), it is clear that the slope has a positive value and ΔH < 0, meaning that the condensation reaction forming Neu5Ac is an exothermic reaction. Since the condensation reaction has a mix of favorable (ΔH < 0) and unfavorable properties (Δ*S* < 0, the system becomes more ordered), this reaction will depend on the temperature and be favorable at low temperatures and less favorable at increasing temperatures (S1 Table). This means that it is advantageous to conduct such condensation reactions at a low temperature to obtain a higher yield of Neu5Ac. This can also be seen from the increasing, less favorable value of Δ*G* with increasing temperature. Additionally, the reduction in reaction rate at lower temperature using a cold active protein as AsNAL, would be less severe. We conducted this experiment with equal amounts of Neu5Ac, ManNAc and pyruvate (5 mM) using the TBA assay. Higher concentrations led to inaccurate results, and our interpretation is that the TBA assay cannot handle high substrate concentrations. This problem has also been pointed out by Brunetti et al. [70]. The equilibrium constants obtained using AsNAL at the three different temperatures are in accordance with previously reported values at 10, 25 and 37°C, as shown in S1 Table.

To drive the equilibrium towards production of Neu5Ac, excess pyruvate and ManNAc are needed to achieve a high yield. Because pyruvate is inexpensive, the equilibrium is moved towards the condensation direction by using higher concentrations of pyruvate. We observed highest yield of Neu5Ac at the ratio of 14:1 (75 mM: 5 mM) after 7.5 h incubation at 23°C (S3A Fig). This is far from inhibitory concentrations of pyruvate (beyond 0.5 M) on NAL activity [76]. A temperature shift from 23°C to 4°C increased the yield by 30% (S3B Fig). The closely similar yield of the temperature-shifted reaction and the one maintained at 4°C indicates that the reaction has reached its equilibrium at that temperature.

### Comparative studies of specific activity and kinetic constants belonging to AsNAL and EcNAL

The His_6_-tag does not affect the activity of the enzyme, because similar results were obtained with both tagged and untagged enzyme. The higher specific activity of AsNAL at all tested temperatures, in both directions, compared to EcNAL, could be a beneficial feature in industrial applications.

The kinetic studies show that the *K*_M_ for AsNAL for the cleavage direction is high compared to other experimental and reported values (Table 1 and S2 Table). The measured value of *K*_M_ for EcNAL is also high compared to other published values for the *E*. *coli* enzyme (S2 Table). This is probably due to different enzyme batches and differences in the type of assay used. The higher *k*_cat_ of AsNAL compared to EcNAL reflects the superior turnover number of AsNAL. A higher reaction rate normally reflects a decreased affinity (higher *K*_M_) as the release of product from the active site is easier. The *K*_M_ of AsNAL is somewhat higher than for EcNAL, indicating a lower affinity for the substrate, which is also quite evident from Fig 4A and 4B. The kinetic data is characteristic for cold adapted enzymes, which often show a higher activity at the cost of a weaker substrate affinity, due to a more flexible active site. It is difficult to compare our *k*_cat_ values with other reported *k*_cat_ values since the method of calculation of *k*_cat_ from V_max_ and units used, are generally not specified. These are therefore not included in S2 Table.

### Structural studies of AsNAL

The overall structure of AsNAL is similar to the structures of the homologous proteins. However, there are differences in the primary structure as shown in Fig 6, resulting in structural features giving rise to the differences we observe in the stability and catalytic rate of the enzymes compared, discussed in more detail below. The structure of AsNAL is providing a basis for redesign of the enzyme to enable cost effective synthesis of Neu5Ac and other sialic acid analogues. The enzyme is also a drug target, and the structure contributes with more structural information that can be utilized in rational drug design.

#### Impact on activity

Class I aldolases catalyze the activation of a ketone donor by forming a Schiff base as an intermediate in the active site, and then adds stereoselectively to the acceptor aldehyde. This stereoselectivity is controlled by the enzyme allowing for highly predictable products in most cases [29]. The reaction mechanism for *N*-acetylneuraminic acid lyase has been described by Barbosa et al. [47]. In the condensation reaction, pyruvate binds first to the catalytic important lysine forming a pyruvate and Lysine-165 Schiff base (*E*. *coli* numbering). The relatively high activity we observe for AsNAL at high pH might be caused by a local depression of the pKa of the essential Lys166 residue.

**Fig 8:**
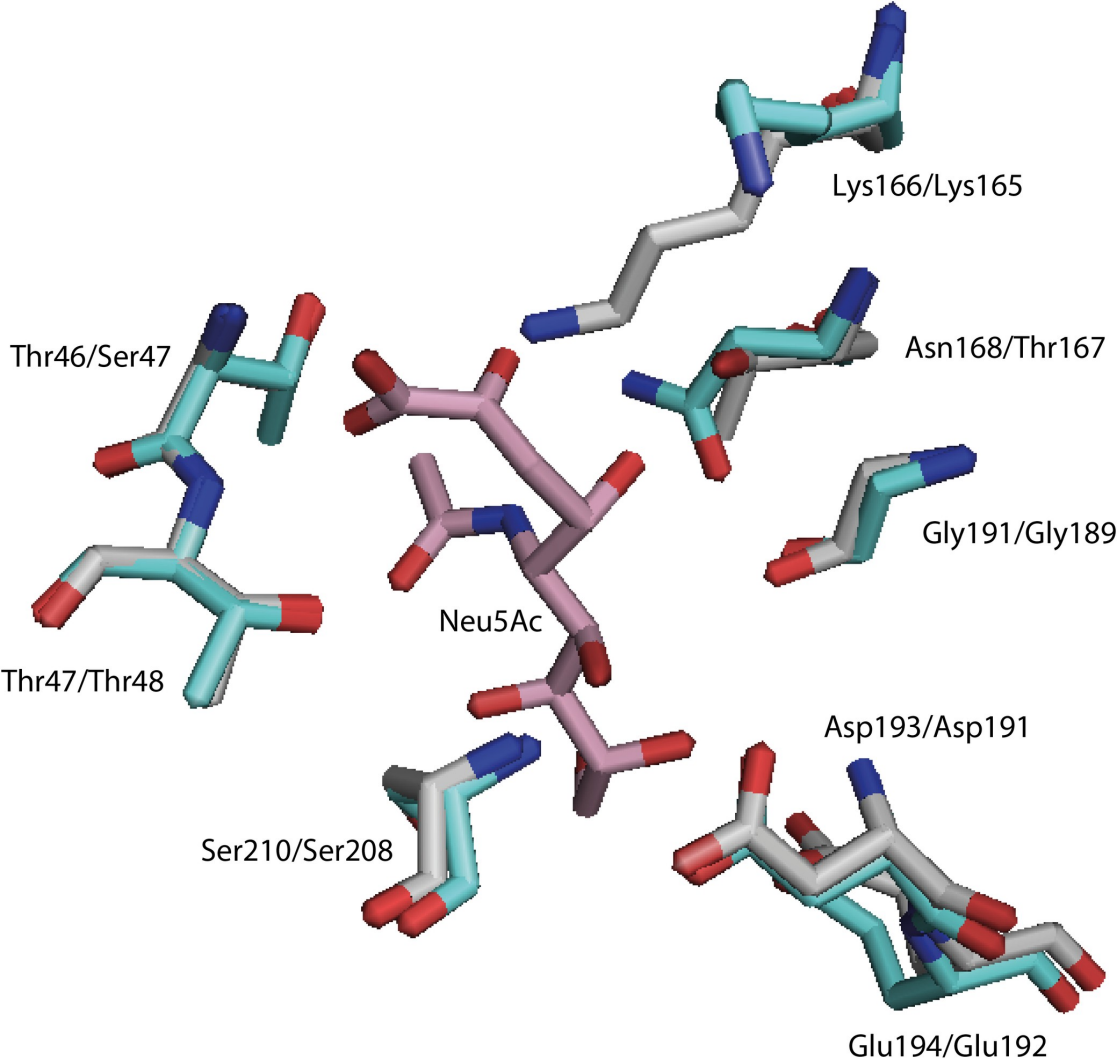
Differences in the substrate binding area between AsNAL (cyan, PDB ID: 5AFD) and EcNAL (grey, PDB ID: 2WNN). The Neu5Ac is from the structure of PmNAL (pink, PDB ID: 4IMF). Residue numbering is shown in the order AsNAL/EcNAL. The EcNAL structure 2WNN is from a wild-type enzyme-pyruvate complex, and the Lys165 is flipped in this structure. Residue Asn168 in AsNAL was shown to be important for the high *k*_cat_ observed.

Daniels et al. [77] further discussed and confirmed the importance of residue Thr167 (*E*. *coli* numbering) in stabilizing the transition state by hydrogen bonding during enzyme catalysis. A mutation removing the possibility to hydrogen bind with substrate (T167A) decreased the *k*_cat_ for Neu5Ac cleavage 4-fold for the EcNAL variant. Replacement with serine (T167S), leaving the H-bonding potential unaffected, left the *k*_cat_ in a similar range. The corresponding residue in AsNAL is Asn168 (Figs 6 and 8). This residue points towards the substrate and is positioned closer to the substrate than what is the case for Thr167 from EcNAL (Fig 8), or for an *in silico* mutation to threonine for AsNAL. Asparagine has a higher H-bonding potential than threonine and might stabilize more central parts of the Neu5Ac molecule (the central N-atom). Our structure of AsNAL shows that Asn168 is able to form two hydrogen bonds with substrates as ManNAc, Neu5Ac and Neu5Gc. Also, *in silico* replacement of EcNAL Thr167 with Asn, results in an extra H-bond to the central N-atom of Neu5Ac. NALs with asparagine in this position are classified into the group 4.4 NALs, as described by Sánchez-Carrón et al. [38]. No other enzymes from this group have been characterized to date. We hypothesized that the substitution to asparagine in this position could explain the high *k*_cat_ observed for AsNAL (Table 1). To further investigate the role of Asn168, two mutants were made, and kinetic characterization of these mutants performed (Table 1). For one of the mutants (N168T), the residue was replaced by the corresponding residue in EcNAL. For the other mutant (N168A), the residue was replaced by alanine, without hydrogen-bonding potential to the substrate. The most striking difference revealed by the kinetic characterization was that both mutants showed considerably lower *k*_cat_ values (Table 1), confirming the importance of this residue in the catalysis. The study by Daniels et al. [77], furthermore showed that mutation of Ser47 in EcNAL had significant impact on the *k*_cat_ value. Mutation to an alanine or cysteine decreased the turnover number, whereas mutation to threonine slightly increased it. The corresponding residue in AsNAL is a threonine. Barbosa et al. [47] also discussed the importance of this residue in ligand binding.

**Fig 9:**
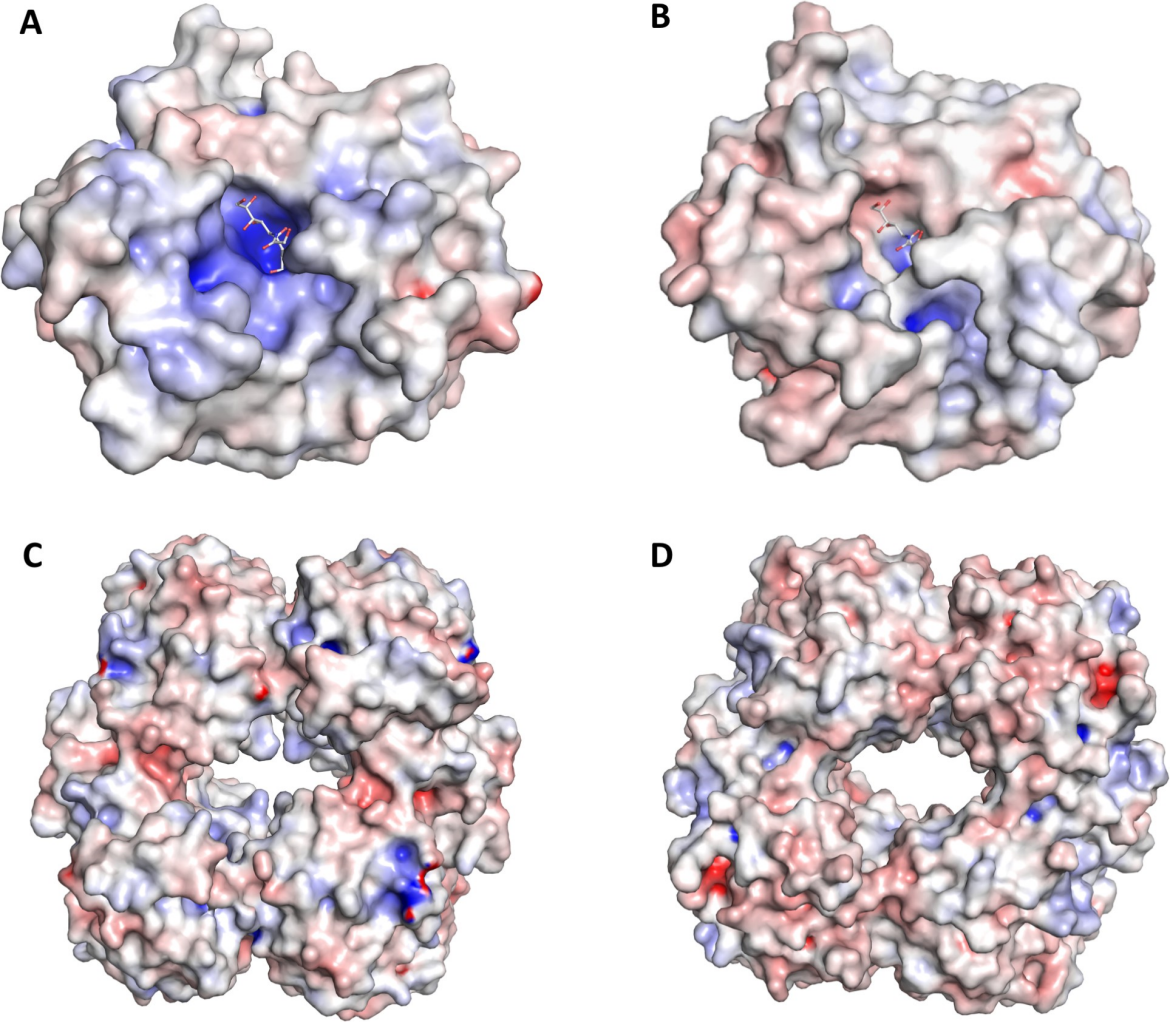
Surface representation of monomers and tetramers of AsNAL (PDB ID: 5AFD) and EcNAL (PDB ID: 2WNN). (A) monomer of EcNAL and (B) monomer of AsNAL with Neu5Ac modelled into the binding site, (C) tetramer of EcNAL and (D) tetramer of AsNAL. The tetramer consists of four chains; A, B, C and D. The surface potential is colored from -10 (red) to 10 (blue) kT/q. Surface potentials were generated using APBS [68] and visualized in PyMol (http://www-pymol.org).

We observed a decreased substrate binding affinity of AsNAL compared to EcNAL (Table 1). The difference in distribution of charged residues was investigated by visualization of the surface potentials of the proteins. Compared to EcNAL (Fig 9A), AsNAL possesses a more negative potential close to the active site (Fig 9B). This might explain the lower substrate affinity observed for AsNAL towards the negatively charged sialic acid. Fig 9A and 9B also show that the binding pocket for AsNAL is narrower compared to EcNAL. The lower substrate affinity might contribute to an easier release of product after the catalytic reaction. The slight increase in substrate affinity seen by substitution to the smaller amino acid alanine for AsNAL N168A reflects its reduced reaction rate, and might be a result of the modified substrate binding pocket. The overall surface potentials of the tetramers of AsNAL and EcNAL were also compared (Fig 9C and 9D). We observe a larger opening in the donut shaped structure of AsNAL compared to EcNAL. This might result in an easier diffusion of substrates to and from the binding site in the interior of the structure, which could also affect the catalytic efficiency that was found to be four times higher for AsNAL compared to EcNAL.

#### Impact on stability

Investigation of structural features that might explain the differences we observe in the stability was performed. The number of hydrogen bonds, salt bridges and the buried surface area between the chains (B+C, A+D and A+C, B+D) were compared for the NAL structures from *A*. *salmonicida* (5AFD), *H*. *influenza* (1F5Z), *S*. *aureus* (4AHQ), *P*. *multocida* (4IMC) and *E*. *coli* (2WNN), and is shown in Table 3. The number of salt bridges and hydrogen bonds is lower for AsNAL compared to the other structures, and the buried surface area is higher. Overall, the lower number of bonds between the chains for AsNAL might explain the reduced temperature stability (77°C) compared to the reported melting temperature for EcNAL (84°C) at neutral pH [46]. The other NALs with structures solved have no reported data for melting temperatures.

**Table 3:** The number of hydrogen bonds, salt bridges and buries surface area between the chains (B+C, A+D and A+C, B+D) for different NAL structures using the PISA server. Cut-off: 4Å.

Bacteria (PDB ID)	H bonds B+C, A+D	H bonds A+C, B+D	Salt bonds B+C, A+D	Salt bonds A+C, B+D	Buried surface area B+C, A+D (average Å)	Buried surface area A+C, B+D (average Å)
*A*. *salmonicida* (5AFD)	7, 7	12, 12	0, 0	8, 8	1500	1172
*H*. *influenza* (1F5Z)	14, 13	11, 12	3, 4	14, 13	1393	1107
*S*. *aureus* (4AHQ)	19, 18	15, 14	6, 6	15, 15	1458	1127
*P*. *multocida* (4IMC)	14, 16	16, 16	4, 4	12, 12	1345	1080
*E*. *coli* (2WNN)	12, 12	13, 12	2, 4	6, 6	1196	1014

An increased global stability was observed at alkaline pH for AsNAL. A further comparison to EcNAL was performed. Most of the residues that become buried upon oligomerization are residues that correspond to each other for the two structures. However, there is a significant difference in the residue type at the interface. There are eight more hydrophobic residues (including Gly) in AsNAL compared to EcNAL (50 versus 42), the number of polar residues is lower (16 compared to 24), whereas the number of charged residues is the same (23). The hydrophobic properties of AsNAL might explain the increased global stability at higher pH, as a change in pH will have less effect on a more hydrophobic protein (fewer exposed bonds that might be interrupted). The pH-range is also broader for AsNAL than for EcNAL.

The total number of residues changing protonation state at pH above 10 compared to pH 7, is similar for AsNAL and EcNAL, both at the interface and exposed areas. A closer investigation and comparison of the location of these residues were performed. There were found some differences that, in addition to the hydrophobic effect, might contribute to the increased stability at alkaline pH. In EcNAL, the catalytically important Tyr137 from one monomer (chain A) lies 4.0 Å apart from Tyr 110 from another monomer (D), and opposite Tyr110 (A) lies 4.1 Å apart from Tyr137 (D). The same is found for Tyr137 (B) that is 4.0 Å apart from Tyr110 (C), and for Tyr110 (B) that is 4.2 Å apart from Tyr137(C). At pH above the pKa of 10.1, the side chain of these residues can be deprotonated. This might result in increased repulsions between the subunits of EcNAL, and thus increased destabilization compared to AsNAL. The corresponding residue in AsNAL is a phenylalanine. Another difference between AsNAL and EcNAL at pH above 10.1 (pKa of the Tyr sidechain) and below pH 12.5 (pKa of the Arg sidechain), is the possibility of increased attraction between the deprotonated Tyr173 and the Arg246 sidechain, between monomers in AsNAL. (Tyr173A- Arg246B, Tyr 173B- Arg246A, Tyr173C-Arg246D and Tyr173D- Arg 246C). In EcNAL, the corresponding Tyr172 has no similar possibility.

In addition, AsNAL contains three more free cysteines than EcNAL. One of these (Cys108) lies close in space to the same residue from another chain of the homo-tetramer (chain A to chain D and chain B to chain C). The distance is 2.6 Å, too far apart from forming a disulfide bridge, which we do not see in the structure. However, at basic conditions above the pKa of the thiolgroups (around 9–10) of cysteine, these will be deprotonated forming a thiolate anion susceptible to oxidation. This might be an explanation of the higher stability observed at alkaline pH compared to EcNAL.

The number of intra-monomeric salt bridges and hydrogen bonds in AsNAL and EcNAL do not differ significantly, and cannot explain the difference we see in stability between the proteins.

## Concluding remarks

This study has described the recombinant production, biochemical characterization and structural determination of the *N*-acetylneuraminate lyase from *A*. *salmonicida*. The protein is a tetramer with high purity and yield after purification and with a tetrameric structure similar to other NALs. Based upon sequence and structural data we constructed a mutant that was important for the high *k*_cat_ observed for Neu5Ac cleavage. We identified interesting enzymatic features of the enzyme, such as high activity and stability at alkaline pH, high activity at low temperature and a higher specific activity compared to the commercially available homologue from *E*. *coli*. We proved that the enzyme can be used at alkaline pH for synthesis of Neu5Ac from the inexpensive precursor *N*-acetylglucosamine. These enzymatic properties make the enzyme a promising biocatalyst, and the data presented provides a framework to guide further exploration of the enzyme. To evaluate the economic viability of its use, we suggest a further optimalization of the application of the enzyme in the synthesis of sialic acid using industrially relevant parameters, such as for example higher substrate concentrations and industrially relevant buffers.

## Supporting information

S1 Fig SDS-PAGE and native PAGE of purified NAL from *A. salmonicida*. (A) Lane 1: Mark12 unstained Standard (Invitrogen), Lane 2: Purified AsNAL (10.2 μg); native PAGE of AsNAL (B) Lane 1: Purified AsNAL (2.75 μg), Lane 2: NativeMark unstained protein Standard (Life technologies).
(TIF)

S2 Fig pH- and temperature-profiles for AsNAL determined by the TBA assay. (A) pH profile for the condensation reaction. (B) pH profile for the cleavage reaction. The buffers used were Sodium phosphate pH 5.5–7.5 (open circles), HEPES pH 6.5–8.0 (open squares), Tris-HCl pH 7.5–9.0 (black squares), and Glycine pH 9.0–11.0 (open triangles). (C) Temperature profile of AsNAL in HEPES buffer pH 8.0 for the condensation (open circles), and cleavage (black circles) reactions after 30 min incubation time. Activity is relative to the highest value measured.
(TIF)

S3 Fig Effect of substrate ratio and temperature shift on Neu5Ac yield. (A) Effect of the [Pyruvate]:[ManNAc] ratio on the yield of Neu5Ac and (B) the increase in Neu5Ac production with shift in temperature from 23°C to 4°C.
(TIF)

S4 Fig Effect of pH on stability and melting temperature of AsNAL. (A) Decrease in activity of AsNAL in condensation direction incubated at different pH for one month at room temperature. Buffers used were Sodium phosphate (pH 6.0–7.0), HEPES (pH 7.0–8.0), Tris-HCl (pH 8.0–9.0) and glycine (pH 9.0–11.0). Decrease in activity was calculated by subtracting the activity of 30^th^ day from activity of 1^st^ day. (B) Effect of pH on *T*_*m*_ of AsNAL. The difference in *T*_*m*_ was calculated by subtracting *T*_*m*_ values obtained in Milli-Q water.
(TIF)

S5 Fig Differential scanning calorimetry (DSC) profile of AsNAL. A melting temperature of 77.5°C at 500 mM NaCl and 50 mM HEPES, pH 7.5 was obtained.
(TIF)

S1 Table Equilibrium constants (*K_c_*) for the condensation direction for NALs (from this study and literature values), in addition with calculated free energy, enthalpy and entropy changes. (PDF)

S2 Table Comparison of features belonging to NALs from different organisms. (PDF)

S1 Appendix PCR primers used in the cloning and cloning procedure. (PDF)

S2 Appendix Datasets used for creation of figures. (PDF)
