# Supplementary figures and images for: Features and structure of a cold active N-acetylneuraminate lyase
Source: PLoS One. 2019 Jun 11;14(6):e0217713. doi: 10.1371/journal.pone.0217713 (PMC6559660; doi:10.1371/journal.pone.0217713)

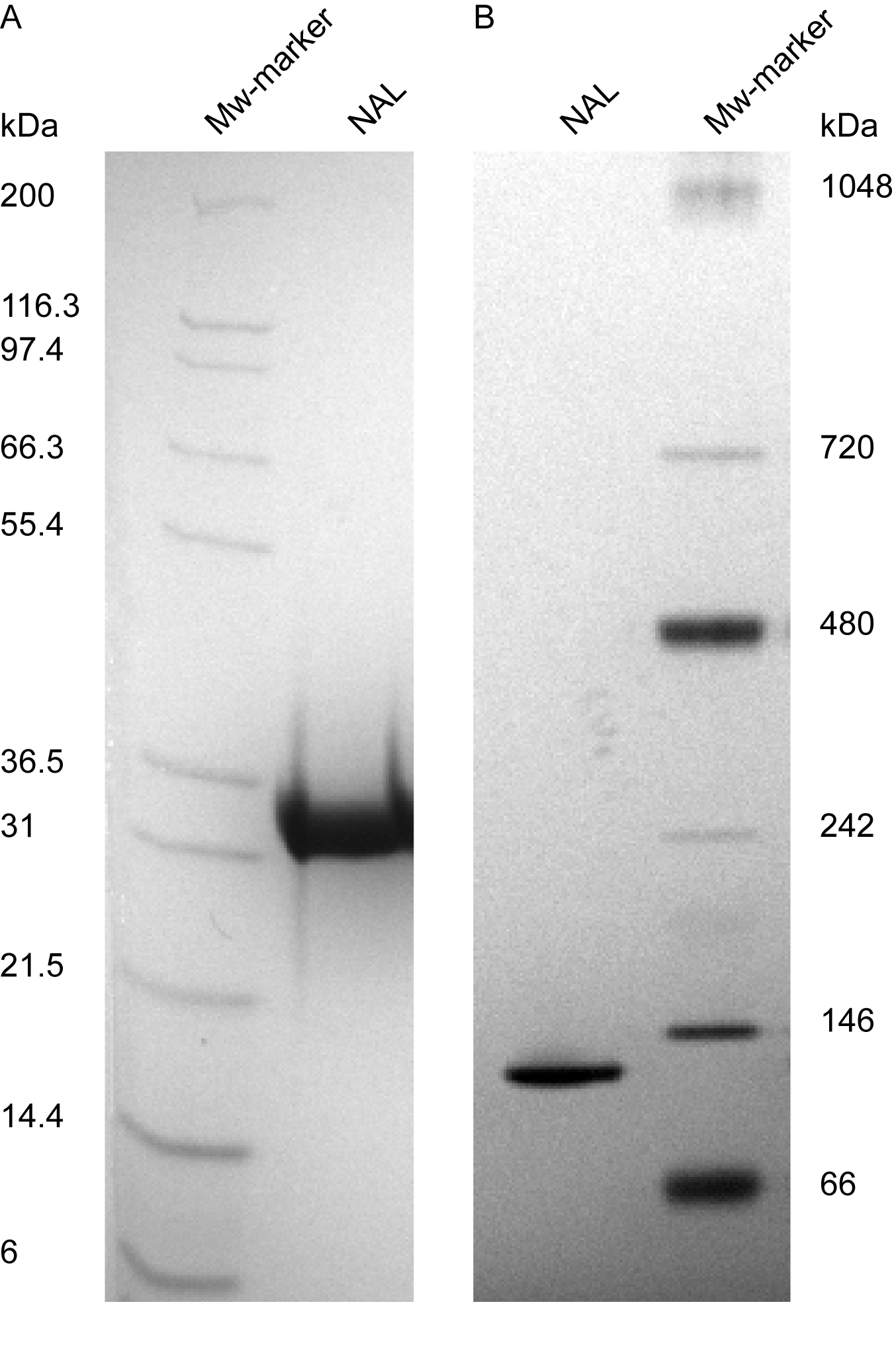

Supplement: S1 Fig — (A) Lane 1: Mark12 unstained Standard (Invitrogen), Lane 2: Purified AsNAL (10.2 μg); native PAGE of AsNAL (B) Lane 1: Purified AsNAL (2.75 μg), Lane 2: NativeMark unstained protein Standard (Life technologies). (TIF) [file pone.0217713.s001.tif]

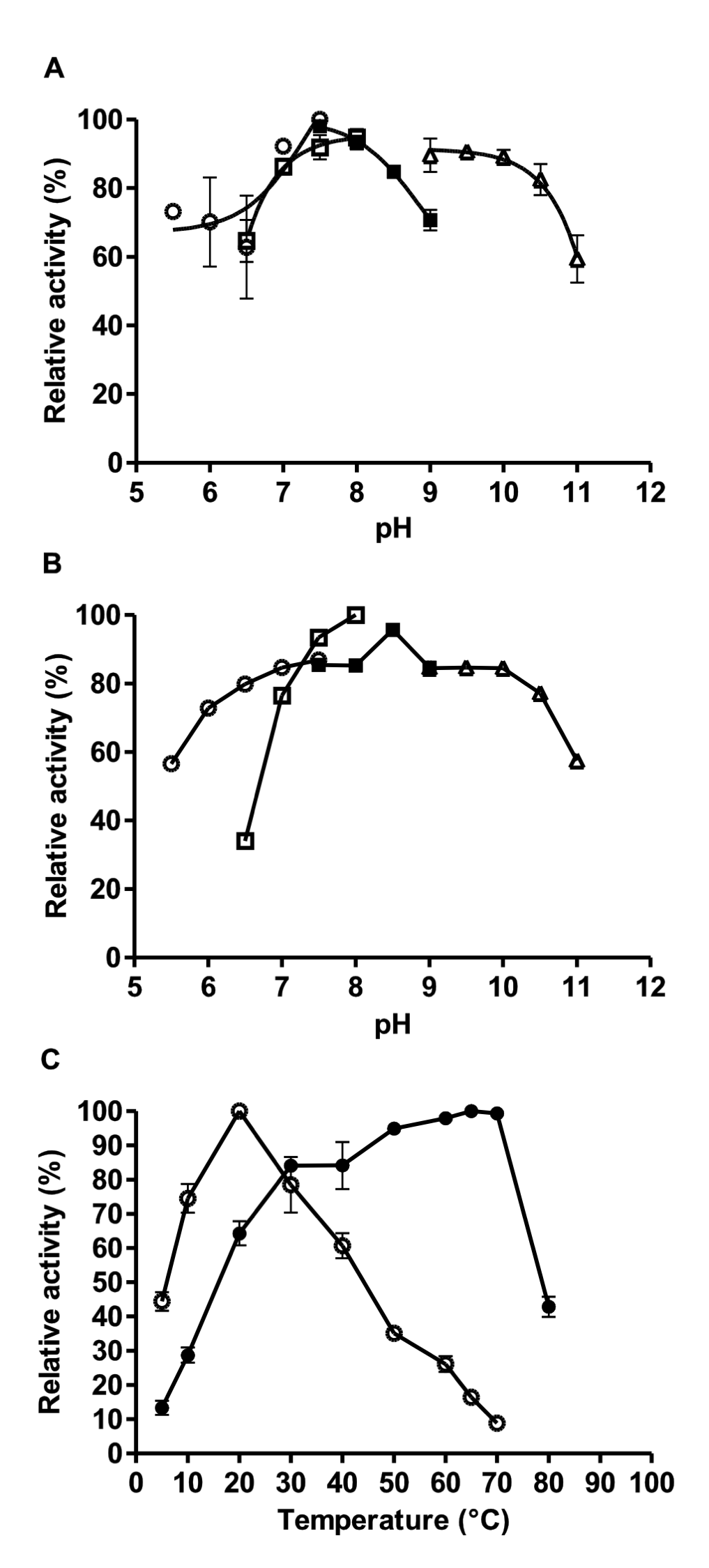

Supplement: S2 Fig — (A) pH profile for the condensation reaction. (B) pH profile for the cleavage reaction. The buffers used were Sodium phosphate pH 5.5–7.5 (open circles), HEPES pH 6.5–8.0 (open squares), Tris-HCl pH 7.5–9.0 (black squares), and Glycine pH 9.0–11.0 (open triangles). (C) Temperature profile of AsNAL in HEPES buffer pH 8.0 for the condensation (open circles), and cleavage (black circles) reactions after 30 min incubation time. Activity is relative to the highest value measured. (TIF) [file pone.0217713.s002.tif]

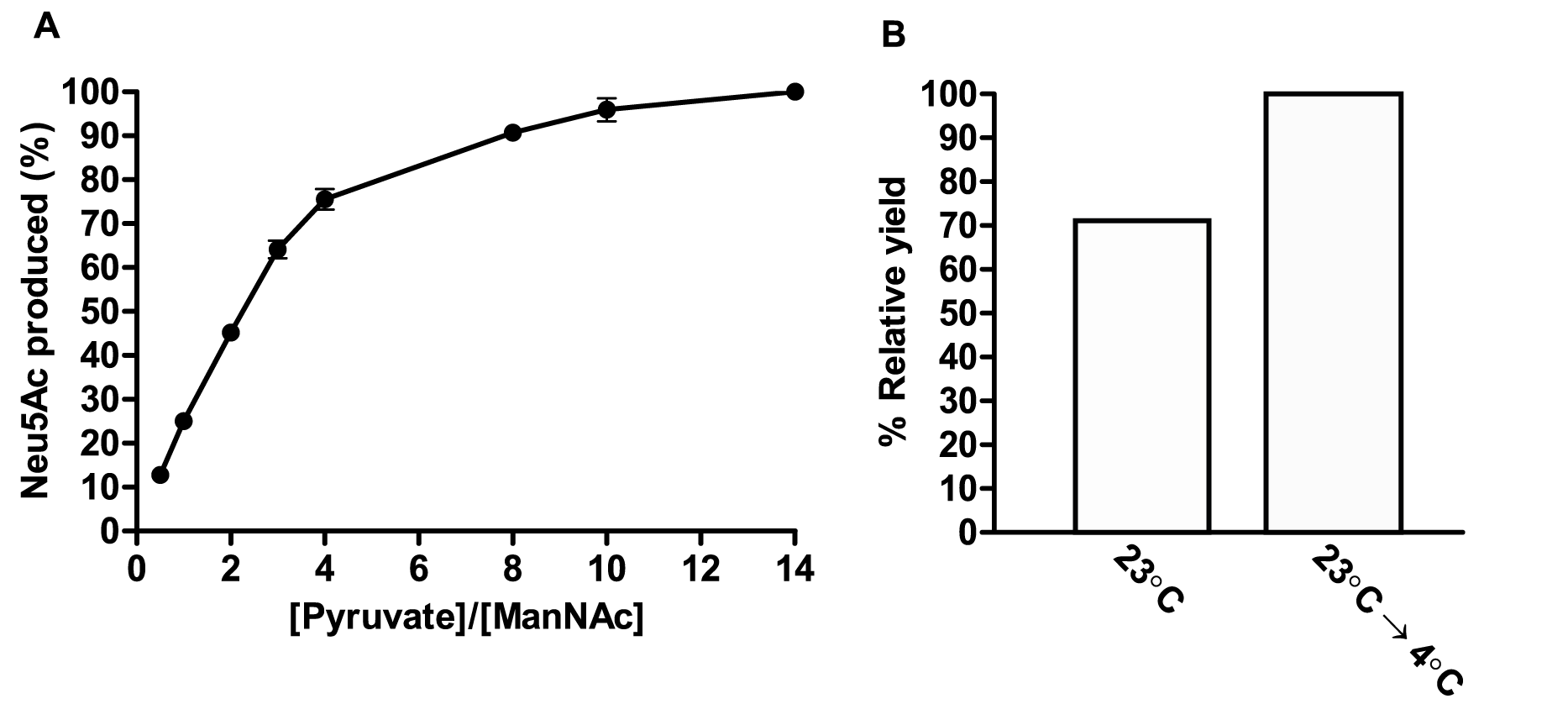

Supplement: S3 Fig — (A) Effect of the [Pyruvate]:[ManNAc] ratio on the yield of Neu5Ac and (B) the increase in Neu5Ac production with shift in temperature from 23°C to 4°C. (TIF) [file pone.0217713.s003.tif]

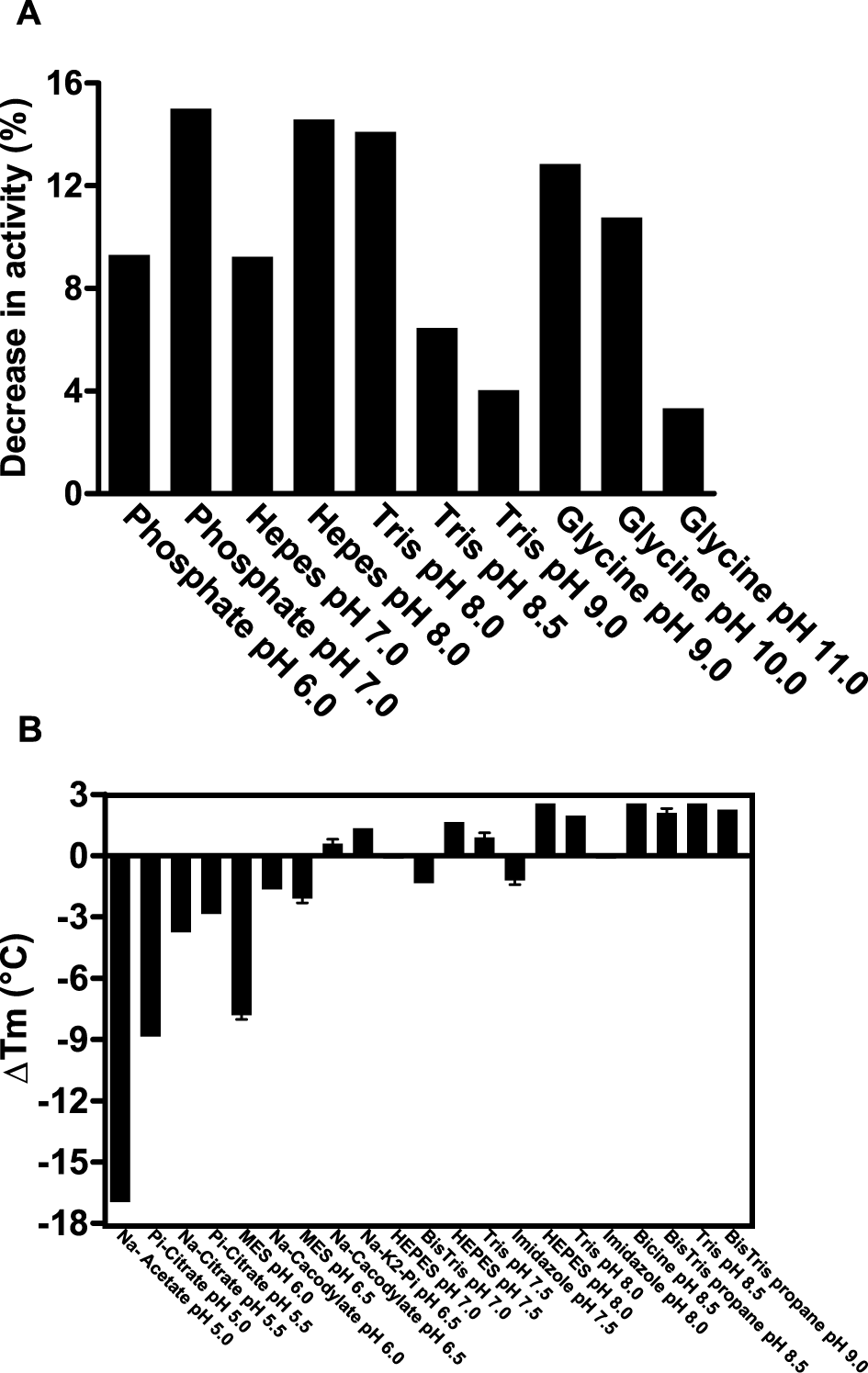

Supplement: S4 Fig — (A) Decrease in activity of AsNAL in condensation direction incubated at different pH for one month at room temperature. Buffers used were Sodium phosphate (pH 6.0–7.0), HEPES (pH 7.0–8.0), Tris-HCl (pH 8.0–9.0) and glycine (pH 9.0–11.0). Decrease in activity was calculated by subtracting the activity of 30th day from activity of 1st day. (B) Effect of pH on Tm of AsNAL. The difference in Tm was calculated by subtracting Tm values obtained in Milli-Q water. (TIF) [file pone.0217713.s004.tif]

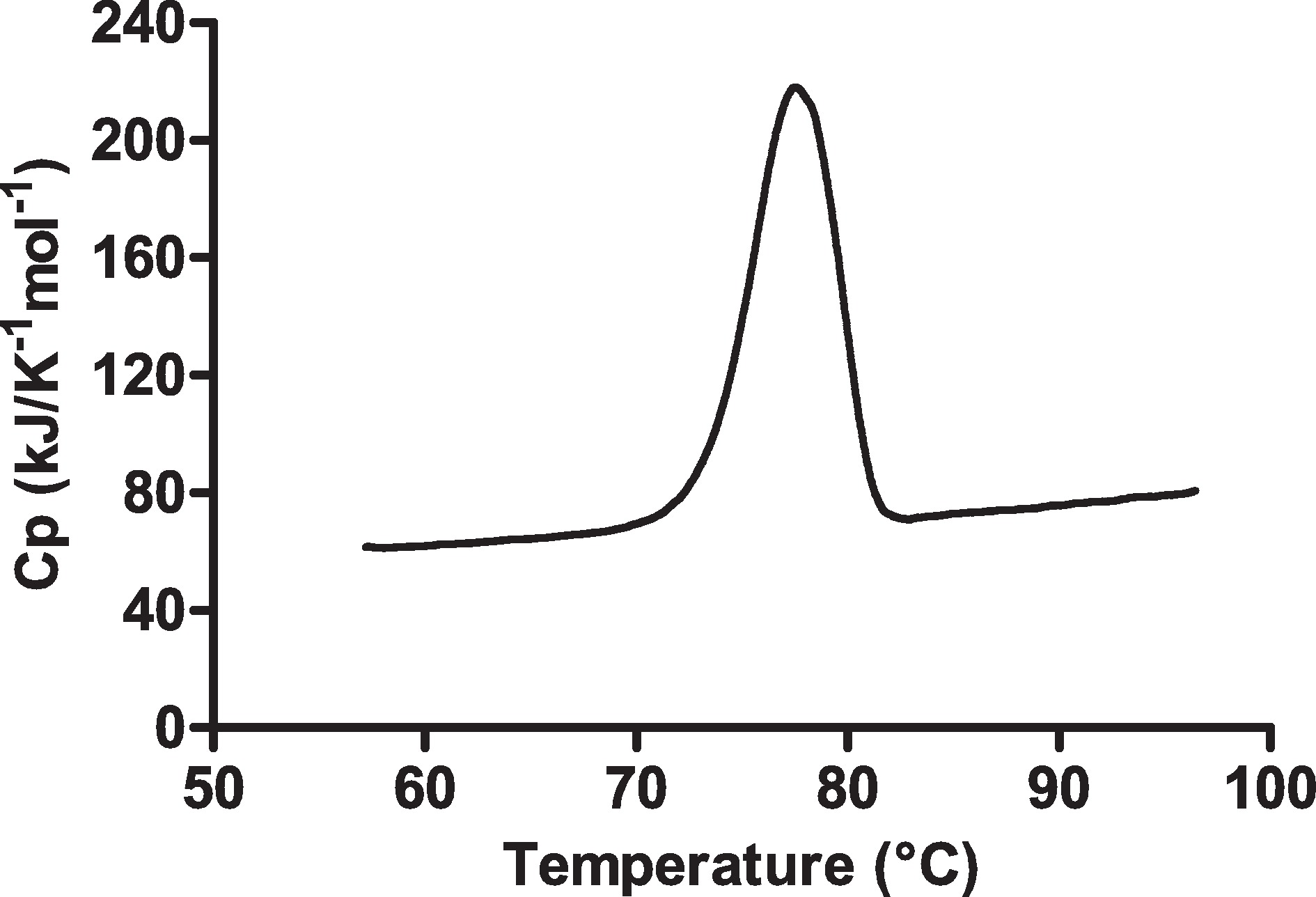

Supplement: S5 Fig — A melting temperature of 77.5°C at 500 mM NaCl and 50 mM HEPES, pH 7.5 was obtained. (TIF) [file pone.0217713.s005.tif]
