# Supplementary material for: Features and structure of a cold active N-acetylneuraminate lyase
Source: PLoS One. 2019 Jun 11;14(6):e0217713. doi: 10.1371/journal.pone.0217713 (PMC6559660; doi:10.1371/journal.pone.0217713)
Supplement: S1 Table — (PDF) [file pone.0217713.s006.pdf]

S2 Table. Equilibrium constants (*K_c*) for the condensation direction for NALs (from this study and literature values), in addition with calculated free energy, enthalpy and entropy changes.

Source for NAL	Temp (°C)	K_c * (M^{-1})	ΔG (kcal/mol)	ΔH (kcal/mol)	TΔS (kcal/mol)	Reference for K_c
A. salmonicida	4	100	-2.6	-11.4	-8.8	This work
Unknown	10	83.1	-2.4	-11.4	-9.0	[1]
A. salmonicida	23	18.9	-2.0	-11.4	-9.4	This work
Unknown	25	28.7	-1.9	-11.4	-9.5	[1]
A. salmonicida	37	9.7	-1.5	-11.4	-9.9	This work
C. perfringens	37	15.6	-1.5	-11.4	-9.9	[2]
Hog kidney cortex	37	10.4	-1.5	-11.4	-9.9	[3]
E. coli	37	12.6	-1.5	-11.4	-9.9	[4]

* K_c = [Neu5Ac]/[ManNAc][Pyruvate]

References

1. Kragl U, Gygax D, Ghisalba O, Wandrey C. Enzymatic Two-Step Synthesis of *N*-Acetyl-neuraminic Acid in the Enzyme Membrane Reactor. Angew Chem Int Ed Engl. 1991; 30 (7): 827-8.
2. Comb DG, Roseman S. The sialic acids. I. The structure and enzymatic synthesis of *N*-acetylneuraminic acid. J Biol Chem. 1960; 235: 2529-37.
3. Brunetti P, Jourdian GW, Roseman S. The sialic acids. III. Distribution and properties of animal *N*-acetylneuraminic aldolase. J Biol Chem. 1962; 237: 2447-53.
4. Uchida Y, Tsukada Y, Sugimori T. Purification and properties of *N*-acetylneuraminate lyase from *Escherichia coli*. J Biochem. 1984; 96 (2): 507-22.
