# Supplementary material for: Features and structure of a cold active N-acetylneuraminate lyase
Source: PLoS One. 2019 Jun 11;14(6):e0217713. doi: 10.1371/journal.pone.0217713 (PMC6559660; doi:10.1371/journal.pone.0217713)
Supplement: S2 Table — (PDF) [file pone.0217713.s007.pdf]

S3 Table. Comparison of features belonging to NALs from different organisms.

Organism	K_M * (Neu5Ac) (mM)	Opt pH (Cond)	Opt pH (Clv)	Opt temp (°C) (Cond)	Opt temp (°C) (Clv)	Reference
A. salmonicida	53.9 ± 6.6	7.5-8.0	8.0-8.5	20	65	This work
E. coli	3.3 ± 0.1	-	6.5-7.0	-	80	[1]
E. coli	3.6	7.7	7.7	-	75	[2]
C. perfringens	1.75	-	7.2	-	-	[3]
E. coli K12	2.5 ± 0.3	7.5-8.0	7.5-8.0	-	-	[4]
P. multocida	4.9 ± 0.7	7.5-8.0	7.5-8.0	-	-	[4]
L. plantarum WCFS1	1.8 ± 0.1	7.0-7.3	7.0-7.3	60	70	[5]
L. antri	1.1 ± 0.1	6.0-7.0	7.0	60	60	[6]
L. sakei	0.3 ± 0.03	6.0	7.0	50	40	[6]
S. carnosus TM300	2 ± 0.3	7.0	7.0	50	60-70	[7]
E. coli	4.4 ± 0.3	-	-	-	-	[8]
E. coli	4.1 ± 0.2	-	-	-	-	[9]
C. perfringens	3.9	-	7.2	-	-	[10]
C. perfringens	3.2	-	7.6	-	65-70	[11]
C. perfringens	2.8	-	7.2	-	-	[12]
C. glutamicum	33.5	8.2-8.4	8.4-8.8	40	40	[13]
Mycoplasma	1.8	7.0	6.0-8.5	65	70	[14]

* All the K_M values have been obtained at 37°C.

References

1. Aisaka K, Igarashi A, Yamaguchi K, Uwajima T. Purification, crystallization and characterization of *N*-acetylneuraminate lyase from *Escherichia coli*. Biochem J. 1991; 276 (Pt 2): 541-6.
2. Uchida Y, Tsukada Y, Sugimori T. Purification and properties of *N*-acetylneuraminate lyase from *Escherichia coli*. J Biochem. 1984; 96 (2): 507-22.
3. DeVries GH, Binkley SB. *N*-acetylneuraminic acid aldolase of *Clostridium perfringens*: Purification, properties and mechanism of action. Arch Biochem Biophys. 1972; 151 (1): 234-42.
4. Li Y, Yu H, Cao H, Lau K, Muthana S, Tiwari VK, et al. *Pasteurella multocida* sialic acid aldolase: a promising biocatalyst. Appl Microbiol Biotechnol. 2008; 79 (6): 963-70.
5. Sanchez-Carron G, Garcia-Garcia MI, Lopez-Rodriguez AB, Jimenez-Garcia S, Sola-Carvajal A, Garcia-Carmona F, et al. Molecular characterization of a novel *N*-acetylneuraminate lyase from *Lactobacillus plantarum* WCFS1. Appl Environ Microbiol. 2011; 77 (7): 2471-8.
6. Garcia-Garcia MI, Gil-Ortiz F, Garcia-Carmona F, Sanchez-Ferrer A. First functional and mutational analysis of group 3 *N*-acetylneuraminate lyases from *Lactobacillus antri* and *Lactobacillus sakei* 23K. PLoS One. 2014; 9 (5): e96976.
7. Garcia Garcia MI, Sola Carvajal A, Garcia Carmona F, Sanchez Ferrer A. Characterization of a novel *N*-acetylneuraminate lyase from *Staphylococcus carnosus* TM300 and its application to *N*-acetylneuraminic acid production. J Agric Food Chem. 2012; 60 (30): 7450-6.
8. Campeotto I, Bolt AH, Harman TA, Dennis C, Trinh CH, Phillips SE, et al. Structural insights into substrate specificity in variants of *N*-acetylneuraminic Acid lyase produced by directed evolution. J Mol Biol. 2010; 404 (1): 56-69.
9. Devenish SR, Gerrard JA. The quaternary structure of *Escherichia coli N*-acetylneuraminate lyase is essential for functional expression. Biochem Biophys Res Commun. 2009; 388 (1): 107-11.
10. Comb DG, Roseman S. The sialic acids. I. The structure and enzymatic synthesis of *N*-acetylneuraminic acid. J Biol Chem. 1960; 235: 2529-37.
11. Kruger D, Schauer R, Traving C. Characterization and mutagenesis of the recombinant *N*-acetylneuraminate lyase from *Clostridium perfringens*: insights into the reaction mechanism. Eur J Biochem. 2001; 268 (13): 3831-9.
12. Nees S, Schauer R, Mayer F. Purification and characterization of *N*-acetylneuraminate lyase from *Clostridium perfringens*. Hoppe-Seyler's Z Physiol Chem. 1976; 357 (6): 839-53.
13. Ji W, Sun W, Feng J, Song T, Zhang D, Ouyang P, et al. Characterization of a novel N-acetylneuraminic acid lyase favoring N-acetylneuraminic acid synthesis. Sci Rep. 2015; 5: 9341.
14. Wang SL, Li YL, Han Z, Chen X, Chen QJ, Wang Y, et al. Molecular Characterization of a Novel *N*-Acetylneuraminate Lyase from a Deep-Sea Symbiotic *Mycoplasma*. Mar Drugs. 2018; 16 (3): 80.
