# Supplementary material for: Features and structure of a cold active N-acetylneuraminate lyase
Source: PLoS One. 2019 Jun 11;14(6):e0217713. doi: 10.1371/journal.pone.0217713 (PMC6559660; doi:10.1371/journal.pone.0217713)
Supplement: S1 Appendix — (PDF) [file pone.0217713.s008.pdf]

S1 Appendix. PCR primers used in the cloning and cloning procedure.

Table A. PCR primers used in the cloning of AsNAL and construction of site-specific mutants.

Primer name	Primer sequence
FPN1	5’-TTCGAAAACCTGTATTTTCAGGGCATGAAAAAGTTAACAGGTTTAATTG-3’
FPN2	5’- GGGGACAAGTTTGTACAAAAAAGCAGGCTTCGAAAACCTG-3’
FPC1	5’-GGAGATAGAACCATGAAAAAGTTAACAGGTTTAATTG-3’
FPC2	5’-GGGGACAAGTTTGTACAAAAAAGCAGGCTTCGAAGGAGATAGAACC-3’
RPN1	5’-GAAAGCTGGGTGTTATTTAAGAAAATTTGCGGCTCTC-3’
RPN2	5’- GGGGACCACTTTGTACAAGAAAGCTGGGTGTTA-3’
RPC1	5’-TTAGTGGTGGTGGTGGTGGTGTTTAAGAAAATTTGCGGCTCTC-3’
RPC2	5’-GGGACCACTTTGTACAAGAAAGCTGGGTCTTAGTGGTGGTGGTGGTGGTG-3’
N168A_F	5'-CGTATAAATCACCGCTGGCAAATTTTAAACCAGATAAGTTAGGAATGCGTT-3’
N168A_R	5’-AACGCATTCCTAACTTATCTGGTTTAAAATTTGCCAGCGGTGATTTATACG-3’
N168T_F	5'-GTATAAATCACCGCTGGTAAATTTTAAACCAGATAAGTTAGGAATGC-3’
N168T_R	5'-GCATTCCTAACTTATCTGGTTTAAAATTTACCAGCGGTGATTTATAC-3’

FPN1 and FPN2 are the forward primers for the N-terminal construct and FPC1 and FPC2 are for the C-terminal construct. RPN1 and RPN2 are the reverse primers for the N-terminal construct and RPC1 and RPC2 for the C-terminal construct. The four last primers are the forward and reverse primers for the N168A and N168T mutants.

Cloning procedure

The gene encoding AsNAL, denoted *nanA*, was initially amplified using the forward primers FPN1 and FPC1 and the reverse primers RPN1 and RPC1, for N-terminally and C-terminally tagged constructs, respectively. The total volume of the PCR reaction was 50 μL containing 1 U Phusion High-Fidelity DNA Polymerase (Finnzymes/Thermo Fischer Scientific, Schwerte, Germany), 1X buffer supplied by the manufacturer, 0.3 mM dNTPs, 0.3 uM of each of the forward and reverse primers and template DNA (genomic DNA of *A. salmonicida*). The PCR was carried out at 98°C for 2 min, followed by 35 cycles of denaturation (98°C for 20 s), annealing (50°C for 20 s), extension (72°C for 20 s) and final extension at 72°C for 7 min. The resultant PCR products were purified from a 1% agarose gel using the Qiaquick Gel Extraction Kit (Qiagen, Hilden, Germany) and subsequently used as templates in a second PCR with the forward primers FPN2 and FPC2 and the reverse primers RPN2 and RPC2 for the N-terminally and C-terminally tagged constructs, respectively. The PCR2 products were purified in the same way as

the first PCR product. The final attB-PCR products were inserted into the destination vector pDEST17 (N-terminal His_6-tag construct) and pDEST14 (C-terminal His_6-tag construct) using BP-and LR-clonase reactions following the “One-Tube Protocol” (Gateway Technology, Invitrogen-Life Technologies, Carlsbad, CA, USA).
