# Supplementary material for: Features and structure of a cold active N-acetylneuraminate lyase
Source: PLoS One. 2019 Jun 11;14(6):e0217713. doi: 10.1371/journal.pone.0217713 (PMC6559660; doi:10.1371/journal.pone.0217713)
Supplement: S2 Appendix — (PDF) [file pone.0217713.s009.pdf]

Datasets

Figure 2

Experiment	Enzyme	Temp (°C)	Time (h)	Sialic acid synthesized (mM)	Sialic acid remaining (mM)	Mean conc. (mM)
A	AsNAL	4	0.0	0.000	5.000000	2.5000000
			0.5	0.5233415	2.444717	1.4840292
			1.0	0.6167076	2.121212	1.3689598
			2.0	0.9156429	2.211302	1.5634724
			3.0	0.9557739	2.082719	1.5192464
			5.0	0.9180999	1.789517	1.3538084
			7.0	0.9459459	1.815725	1.3808354
			18.0	1.127764	1.915643	1.5217035
			19.5	1.048321	1.672400	1.3603605
			24.0	1.107289	1.524979	1.316134
			26.0	1.128583	1.467649	1.298116
			43.0	1.249795	1.737920	1.4938575
B	AsNAL	23	0.0	0.0	5.000000	2.5000000
			0.5	0.3374283	1.857494	1.09
			1.0	0.2989353	1.289926	0.7944306
			2.0	0.3226863	0.9770679	0.6498771
			3.0	0.3243243	0.6453726	0.4848484
			5.0	0.3046683	0.4578215	0.3812449
			7.0	0.3079443	0.4209664	0.3644553
			18.0	0.3906634	0.4897625	0.4402129
			19.5	0.3603604	0.5036855	0.4320229
			24.0	0.3366093	0.4627355	0.3996724
			26.0	0.3235053	0.4701065	0.3968059
			43.0	0.3972154	0.5986896	0.4979525
C	AsNAL	37	0.0	0.0	5.000000	2.500000
			0.25	0.1089271	0.9074529	0.50819
			0.5	0.1056511	0.4627355	0.2841933
			1.0	0.1072891	0.2145782	0.1609336
			2.0	0.1081081	0.1605242	0.1343161
			3.0	0.1367731	0.1785422	0.1576576
			4.0	0.1375921	0.1728092	0.1552006
			5.0	0.1547912	0.1908272	0.1728092
			6.0	0.1531532	0.2031122	0.1781327
			7.0	0.1572482	0.2137592	0.1855037
			8.0	0.1638002	0.2194922	0.1916462
			10.0	0.1728092	0.2334152	0.2031122
			13.0	0.2252252	0.3104013	0.2678132
			22.0	0.2170352	0.4717445	0.3443898
			24.0	0.2358722	0.2055692	0.2207207
			27.0	0.1294021	0.1654382	0.1474201

Figure 3

Experiment	Enzyme	Direction	Temp (°C)	% Relative activity	Mean (% Relative activity)	% STDEV
A	AsNAL	Condensation	4	66.13217	67.34847	2.314948
				65.8952		
				70.01803		
			23	100	99.95423	0.061519
				99.8843		
				99.9784		
			37	84.52381	78.89148	4.922249
				75.41485		
				76.7358		
	EcNAL	Condensation	4	19.09375	22.50241	3.359343
				22.60332		
				25.81016		
			23	38.21422	34.65845	3.300055
				31.69408		
				34.06706		
			37	36.90372	34.44701	2.241775
				32.51132		
				33.92624		
B	AsNAL	Cleavage	4	60.50553	60.27167	0.617791
				59.57109		
				60.73839		
			23	88.46761	88.86319	0.368881
				89.19778		
				88.92418		
			37	100	99.94157	0.054506
				99.8921		
				99.9326		
	EcNAL	Cleavage	4	33.60021	34.61274	0.877594
				35.15446		
				35.08355		
			23	51.30984	51.3656	0.10454
				51.30111		
				51.48622		
			37	63.8574	64.1568	0.260293
				64.28366		
				64.32934		

Figure 4

Experiment	Enzyme	Substrate conc. (mM)	Vmax1	Vmax2	Vmax3	Mean Vmax (µM/min)	STDEV
A	AsNAL	1	0.5901318	0.4792662	0.5480096	0.539135867	0.055963
		5	1.937789	2.209056	2.155982	2.100942333	0.1437653
		15	5.922379	5.569396	5.818759	5.770178	0.1814369
		30	9.835868	8.959726	9.65053	9.482041333	0.4617332
		45	12.13439	11.9322	11.32565	11.79741333	0.4208808
		60	15.47788	13.64978	14.15524	14.42763333	0.944
		75	17.30978	14.98463	15.44798	15.91413	1.2306716
		90	17.32486	14.99129	16.00222	16.10612333	1.1702496
B	AsNAL N168A	1	0.7485113	0.6104347	0.5598881	0.639611367	0.0976378
		5	2.291447	1.858431	1.914875	2.021584333	0.2354058
		15	5.404276	5.242527	5.042026	5.229609667	0.1814701
		30	7.948457	7.409293	7.758907	7.705552333	0.2735132
		45	9.325852	9.426946	9.165788	9.306195333	0.131684
		60	10.57267	10.65691	10.76643	10.66533667	0.0971545
		75	11.52463	11.6173	11.48251	11.54148	0.0689567
		90	12.51871	13.14212	13.26849	12.97644	0.4014099
C	AsNAL N168T	1	0.7202051	0.6965325	0.8257633	0.7475003	0.0688035
		5	2.523961	2.628424	3.051331	2.734572	0.2792496
		15	7.350322	7.276186	7.598843	7.408450333	0.1690002
		30	11.75209	12.13119	12.45132	12.11153333	0.3500292
		45	15.76212	15.79582	16.43608	15.99800667	0.3797566
		60	18.45794	17.81768	19.90695	18.72752333	1.0704058
		75	19.83113	19.57839	21.03582	20.14844667	0.7788087
		90	21.76032	23.20932	21.50759	22.15907667	0.9182736
D	EcNAL	1	0.7882746	0.6571904	0.5989775	0.681480833	0.0969581
		5	2.546202	2.291784	2.609385	2.482457	0.1681225
		15	6.747385	5.929372	5.294169	5.990308667	0.7285219
		30	7.877607	7.183434	7.150578	7.403873	0.4105945
		45	9.688946	8.686438	8.20119	8.858858	0.7587167
		60	11.53188	10.79895	9.274126	10.53498533	1.1517905
		75	12.83042	11.28875	10.26939	11.46285333	1.2893613
		90	14.42146	13.21676	12.6439	13.42737333	0.9073028

Figure 5

Experiment	Enzyme	Time (h)	% Conversion	Mean (% conversion)	% STDEV
A	AsNAL	0.5	25.97872	24.14903667	1.922356
			22.14578		
			24.32261		
		1	26.57658	27.09944	0.516113
			27.60853		
			27.11321		
		1.5	29.24652	32.01517	2.784508
			34.81527		
			31.98372		
		2.5	30.05733	31.54939	1.515678
			33.08763		
			31.50321		
	EcNAL	0.5	5.76577	6.303253333	0.532424
			6.83047		
			6.31352		
		1	11.3104	12.50042667	1.25142
			13.80532		
			12.38556		
		1.5	20.10647	20.80565333	0.704397
			21.51515		
			20.79534		
		2.5	30.18968	27.74022667	2.621777
			24.97477		
			28.05623		
**Experiment**	**Enzyme**	**Time (h)**	**% Conversion**	**Mean (% conversion)**	**% STDEV**
B	AsNAL	12	4.29975	4.217523333	0.412887
			4.58311		
			3.76971		
		24	8.07979	8.364253333	0.285931
			8.36134		
			8.65163		
		36	13.61179	13.20229	0.4095
			12.79279		
			13.20229		
		48	18.74693	18.60675	0.177936
			18.40657		
			18.66675		
	EcNAL	12	0.63012	0.61818	0.100434
			0.51231		
			0.71211		
		24	1.44859	1.312406667	0.12755
			1.29289		
			1.19574		

		36	1.65299	1.837166667	0.258233
			2.13234		
			1.72617		
		48	3.15392	2.936786667	0.189314
			2.80632		
			2.85012		

S2 Fig

**Experiment**	A				
**Enzyme**	AsNAL				
**Direction**	Condensation				
**Temp (°C)**	23				
**Buffer**	**% Relative activity 1**	**% Relative activity 2**	**% Relative activity 3**	**Mean (% Relative activity)**	**% STDEV**
Phosphate pH 5.5	74.87277354	71.80589681	72.89308176	73.19058403	1.5549321
Phosphate pH 6.0	81.55216285	72.85012285	56.10062893	70.16763821	12.936071
Phosphate pH 6.5	50.92239186	57.89312039	79.65408805	62.8232001	14.986889
Phosphate pH 7.0	93.47964377	90.26412776	92.79874214	92.18083789	1.6944734
Phosphate pH 7.5	100	100	100	100	0
Hepes pH 6.5	71.43765903	63.20638821	59.43396226	64.69266983	6.1383189
Hepes pH 7.0	88.7086514	84.18304668	85.94339623	86.27836477	2.2813214
Hepes pH 7.5	92.58905852	88.05282555	95.06289308	91.90159239	3.5552382
Hepes pH 8.0	95.89694656	92.10687961	96.44654088	94.81678902	2.3628839
Tris pH 7.5	99.80916031	96.00737101	98.42767296	98.08140142	1.9244034
Tris pH 8.0	94.97455471	91.09336609	93.77358491	93.2805019	1.9870215
Tris pH 8.5	83.04707379	84.61302211	86.76100629	84.80703406	1.864552
Tris pH 9.0	73.05979644	71.77518428	67.32704403	70.72067491	3.0083393
Glycine pH 9.0	93.73409669	90.75552826	84.24528302	89.57830266	4.8527097
Glycine pH 9.5	91.7302799	88.45208845	91.69811321	90.62682719	1.8834477
Glycine pH 10.0	91.09414758	86.85503686	89.05660377	89.0019294	2.1200842
Glycine pH 10.5	87.37277354	81.66461916	78.4591195	82.4988374	4.5150025
Glycine pH 11.0	66.57124682	58.75307125	52.86163522	59.39531776	6.877334

**Experiment**	B				
**Enzyme**	AsNAL				
**Direction**	Cleavage				
**Temp (°C)**	23				
**Buffer**	**% Relative activity 1**	**% Relative activity 2**	**% Relative activity 3**	**Mean (% Relative activity)**	**% STDEV**
Phosphate pH 5.5	55.85447092	56.67693888	57.32614832	56.61918604	0.7375365
Phosphate pH 6.0	73.20010249	72.98407807	72.38901719	72.85773258	0.4200443
Phosphate pH 6.5	80.32282859	79.12172573	80.13856813	79.86104082	0.6468602

Phosphate pH 7.0	84.24289008	85.36209553	84.52655889	84.71051484	0.5818376
Phosphate pH 7.5	86.44632334	87.18541346	86.88734924	86.83969535	0.3718423
Hepes pH 6.5	34.71688445	34.72008218	32.69181422	34.04292695	1.170099
Hepes pH 7.0	77.55572636	76.27118644	75.82755966	76.55149082	0.8975344
Hepes pH 7.5	93.44094286	93.57986646	93.27687965	93.43256299	0.1516671
Hepes pH 8.0	100	100	100	100	0
Tris pH 7.5	85.72892647	85.46481767	85.09109571	85.42827995	0.3204813
Tris pH 8.0	85.26774276	85.46481767	85.2450603	85.32587358	0.1208624
Tris pH 8.5	95.79810402	95.40318439	95.86861688	95.68996843	0.2508522
Tris pH 9.0	84.16602613	84.82280431	84.83448807	84.60777284	0.3826085
Glycine pH 9.0	84.75531642	84.48895737	84.42391583	84.55606321	0.1755961
Glycine pH 9.5	83.93543428	84.20647149	85.91224018	84.68471532	1.0716708
Glycine pH 10.0	85.19087881	84.30919363	84.16730819	84.55579354	0.5545564
Glycine pH 10.5	77.24827056	77.81201849	76.6743649	77.24488465	0.5688344
Glycine pH 11.0	56.80245965	58.24345146	57.50577367	57.51722826	0.7205642

**Experiment**	C				
**Enzyme**	AsNAL				
**Direction**	Condensation				
**Temp °C**	**% Relative activity 1**	**% Relative activity 2**	**% Relative activity 3**	**Mean (% Relative activity)**	**% STDEV**
5	43.05373526	42.65944645	47.53989362	44.41769177	2.7110836
10	75.6225426	69.91576414	78.19148936	74.5765987	4.2358477
20	100	100	100	100	0
30	70.11795544	79.30204573	86.17021277	78.53007131	8.0539245
40	62.8440367	62.87605295	56.51595745	60.74534903	3.6627955
50	35.77981651	33.21299639	36.30319149	35.09866813	1.6538739
60	24.9672346	24.66907341	28.78989362	26.14206721	2.2979259
70	15.92398427	15.46329723	18.01861702	16.46863284	1.3619458
80	10.55045872	8.423586041	7.646276596	8.873440451	1.5034444
**Direction**	Cleavage				
**Temp °C**	**% Relative activity 1**	**% Relative activity 2**	**% Relative activity 3**	**Mean (% Relative activity)**	**% STDEV**
5	14.83697278	14.27714457	10.95066185	13.3549264	2.100885
10	26.6826204	28.43431314	31.04693141	28.72128832	2.1962624
20	65.45019444	60.34793041	67.14801444	64.31537976	3.5392286
30	83.78701765	84.10317936	84.44645006	84.11221569	0.3298091
40	80.46664672	92.11157768	79.96389892	84.18070778	6.8729333
50	95.1839665	96.13077385	93.56197353	94.95890462	1.2991048
60	97.81633264	97.99040192	97.95427196	97.92033551	0.0918629
65	100	100	100	100	0
70	99.25216871	99.34013197	99.45848375	99.35026148	0.1035298
80	41.63924619	40.67186563	46.20938628	42.84016603	2.9576494

S3A Fig

Ratio (Pyruvate: ManNAc)	% Conversion (Parallell 1-Blank)	% Conversion (Parallell 2-Blank)	% Conversion (Parallell 3-Blank)	% STDEV	Mean (%Conversion)
0.5	10.89259	12.67716535	14.70814132	1.90910268	12.75929789
1	24.58396	26.41732283	24.03993856	1.2456006	25.01374169
2	44.51589	46.49606299	44.58525346	1.12376672	45.19906716
3	63.69138	66.2992126	6.3655914	2.00132833	64.1187269
4	73.44932	78.03149606	75.19201229	2.31286229	75.55760919
8	90.24206	90.47244094	91.47465438	0.65533749	90.7297176
10	93.04085	98.11023622	96.62058372	2.60551604	95.92388905
14	99.46342	100	99.8911232	0.28363776	100

S3B Fig

Temp (°C)	Absorbance (OD549-Blank)	Mean absorbance (OD549-Blank)	%Relative activity	% STDEV
23	2.696	2.668	71.52176	0.809561
	2.672			
	2.636			
23 → 4	3.676	3.730333	100	1.488789
	3.728			
	3.787			

S4 Fig

Experiment A				
pH	% Relative activity at day 1	% Relative activity after 1 month	Δdrop % activity after 1 month	% STDEV
Phosphate pH 6.0	87.91666667	78.61111111	9.305555556	3.951092682
Phosphate pH 7.0	100	85	15	1.178511302
Hepes pH 7.0	85.13888889	75.90277778	9.236111111	2.258813329
Hepes pH 8.0	100	85.41666667	14.58333333	2.160604054
Tris pH 8.0	97.22222222	83.125	14.09722222	0.883883476
Tris pH 8.5	84.58333333	78.125	6.458333333	2.848068982
Tris pH 9.0	72.77777778	68.75	4.027777778	1.964185503
Glycine pH 9.0	96.11111111	83.26388889	12.84722222	1.865976228
Glycine pH 10.0	88.19444444	77.43055556	10.76388889	1.080302027
Glycine pH 11.0	51.25	47.91666667	3.333333333	1.754348322

Experiment B				
Buffers	Mean Tm (°C)	STDEV	No buffer mean Tm (°C)	ΔTm (°C)
Blank	73.15	0.212132	73.15	0
Sod. Acetate pH 5.0	56.2	0	73.15	16.95
Pi-Citrate pH 5.0	64.3	0	73.15	8.85
Na-Citrate pH 5.5	69.4	0	73.15	3.75

Pi-Citrate pH 5.5	70.3	0	73.15	2.85
MES pH 6.0	65.35	0.212132	73.15	7.8
Na-Cacodylate pH 6.0	71.5	0	73.15	1.65
MES pH 6.5	71.05	0.212132	73.15	2.1
Na-Cacodylate pH 6.5	73.75	0.212132	73.15	-0.6
Na-K2-Pi pH 6.5	74.5	0	73.15	-1.35
HEPES pH 7.0	73	0	73.15	0.15
BisTris pH 7.0	71.8	0	73.15	1.35
HEPES pH 7.5	74.8	0	73.15	-1.65
Tris pH 7.5	74.05	0.212132	73.15	-0.9
Imidazole pH 7.5	71.95	0.212132	73.15	1.2
HEPES pH 8.0	75.7	0	73.15	-2.55
Tris pH 8.0	75.1	0	73.15	-1.95
Imidazole pH 8.0	73	0	73.15	0.15
Bicine pH 8.5	75.7	0	73.15	-2.55
BisTris propane pH 8.5	75.25	0.212132	73.15	-2.1
Tris pH 8.5	75.7	0	73.15	-2.55
BisTris propane pH 9.0	75.4	0	73.15	-2.25
